# The 1α-hydroxy-A-rings of norditerpenoid alkaloids are twisted-boat conformers†

**DOI:** 10.1039/d0ra03811c

**Published:** 2020-05-18

**Authors:** Ziyu Zeng, Ashraf M. A. Qasem, Gabriele Kociok-Köhn, Michael G. Rowan, Ian S. Blagbrough

**Affiliations:** Department of Pharmacy and Pharmacology, University of Bath Bath BA2 7AY UK prsisb@bath.ac.uk; Material and Chemical Characterisation (MC^2^), University of Bath Bath BA2 7AY UK

## Abstract

The skeletal conformations of naturally occurring norditerpenoid alkaloids fix their substituent functional groups in space, thereby directing their bioactivities. Solution conformations of the A-rings of 4 selected norditerpenoid alkaloid free bases: mesaconitine, karacoline (karakoline), condelphine, and neoline (bullatine B), were analysed by NMR spectroscopy and single-crystal X-ray crystallography. They adopt twisted-chair, twisted-boat, twisted-boat, twisted-boat conformations, respectively. That the A-ring is stabilised in a boat conformer by an intramolecular H-bond from 1α-OH to the *N*-ethyl tertiary amine is also confirmed in the condelphine single crystal data. The conformations are a result of through-space repulsion between 12-H_e′_ and atoms attached to C1 (in the equatorial positions). This causes the A-rings with 1α-OR always to be twisted whether in a chair or a boat conformation. The impact of these studies is in providing a detailed understanding of the shape of the A-ring of these important biologically active natural product alkaloids.

## Abstract 2

The skeletal conformations of naturally occurring norditerpenoid alkaloids fix their substituent functional groups in space, thereby directing their bioactivities.

## Graphical abstract

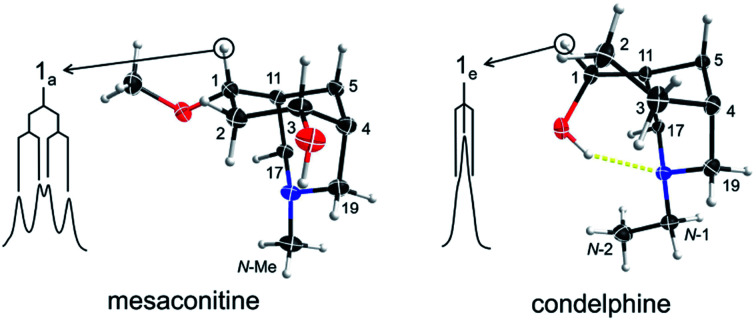

## 1 Introduction

**Fig. 1:**
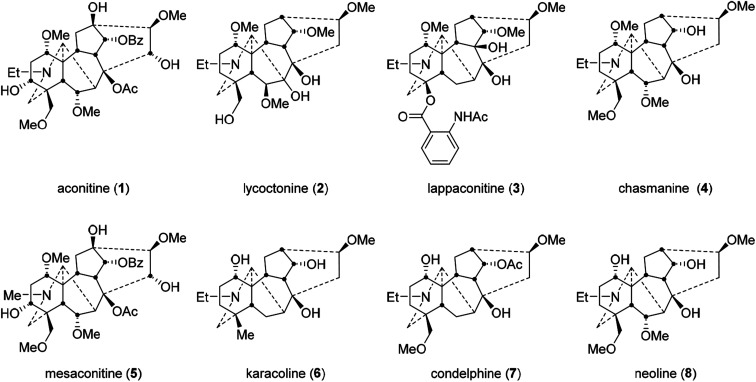
Selected norditerpenoid alkaloids.

The unambiguous structural identification of naturally occurring norditerpenoid alkaloids with 6 fused and bridged rings is complicated. Evaluation of their structure in terms of both conformation and configuration is important as 3D orientation is a key parameter for controlling biological activity. The positions of certain functional groups were not initially assigned accurately.^1–4^ With application of single crystal X-ray diffraction (SXRD), several important norditerpenoid alkaloids were eventually evaluated, *e.g.* aconitine (1, Fig. 1) was first reported in 1833,^5^ and its structure was confirmed in 1959;^6,7^ lycoctonine (2) was first reported in 1865,^8^ and its structure was confirmed in 1956;^9^ lappaconitine (3) was first reported in 1895,^10,11^ and its structure was confirmed in 1969.^12,13^ The orientational assignments of oxygenated groups attached to C1 of several norditerpenoid alkaloids were revised unambiguously using SXRD data, *e.g.* aconitine (1), lycoctonine (2), and chasmanine (4).^14–20^ Solution conformation studies can provide detail of the bioactive conformations in biological fluids of these pharmacologically important norditerpenoid alkaloids. Specifically, we are studying the effects of the C1-α-oxygenated substituents on the A-rings in four selected norditerpenoid alkaloids (5–8).

## 2 Results and discussion

Complete and unambiguous NMR spectroscopic data of mesaconitine (5, Fig. 1), karacoline (6, karakoline), condelphine (7), and neoline (8, bullatine B) were obtained (Fig. S1–S26†). Some literature assignments of ^13^C NMR signals of mesaconitine (5) and condelphine (7) have been revised supported by 2D NMR data (Tables S1–S4†). Due to repulsion between 1α-OMe (in the equatorial position)/12-H_e′_ proposed by Pelletier and co-workers,^13,21,22^ the A-ring of norditerpenoid alkaloid free bases with 1α-OMe adopting chair conformations are twisted. This significant proximity decreases when the A-rings adopt boat conformations as the O-atom flips to the axial position and therefore away from 12-H_e′_, *e.g.* norditerpenoid alkaloid (with 1α-OMe/OH) salts and norditerpenoid alkaloid free bases with 1α-OH, which may lead to the A-rings adopting true-boat (not twisted) conformations.

**Fig. 2:**
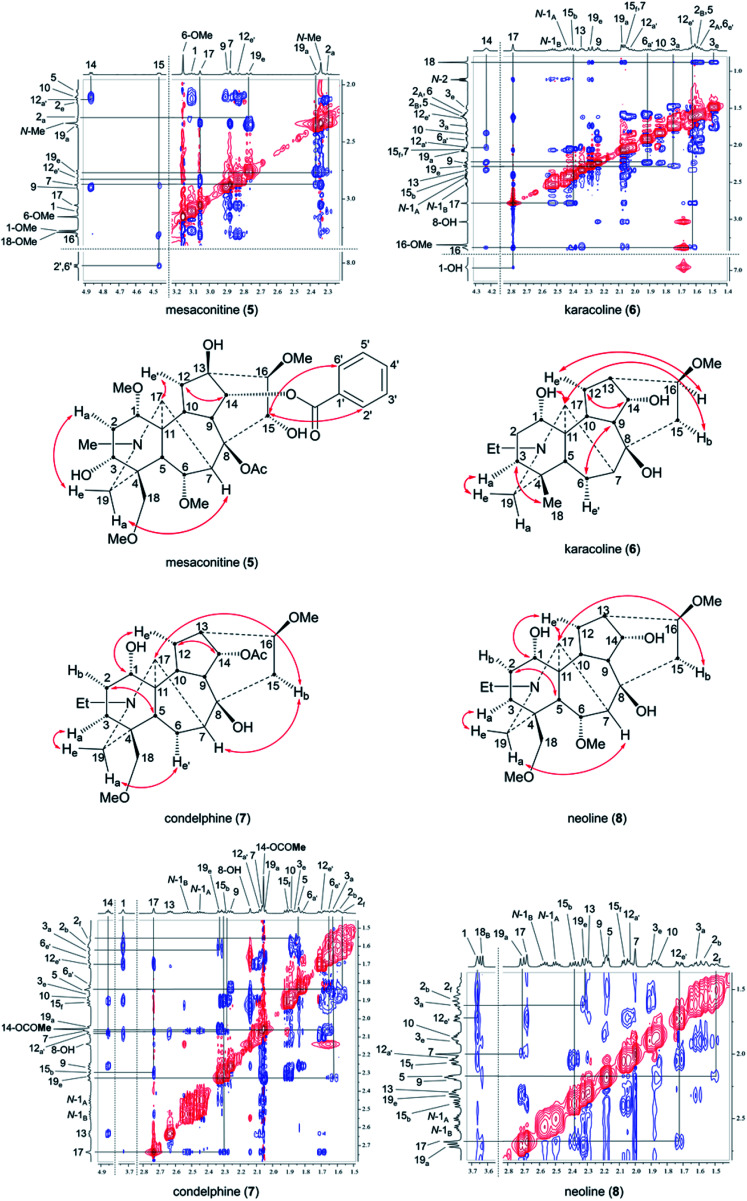
Key NOESY correlations of the selected norditerpenoid alkaloids. Orientation label: a = axial, e = equatorial, b = bowsprit, f = flagpole, a′ = pseudo-axial, e′ = pseudo-equatorial.

**Fig. 3:**
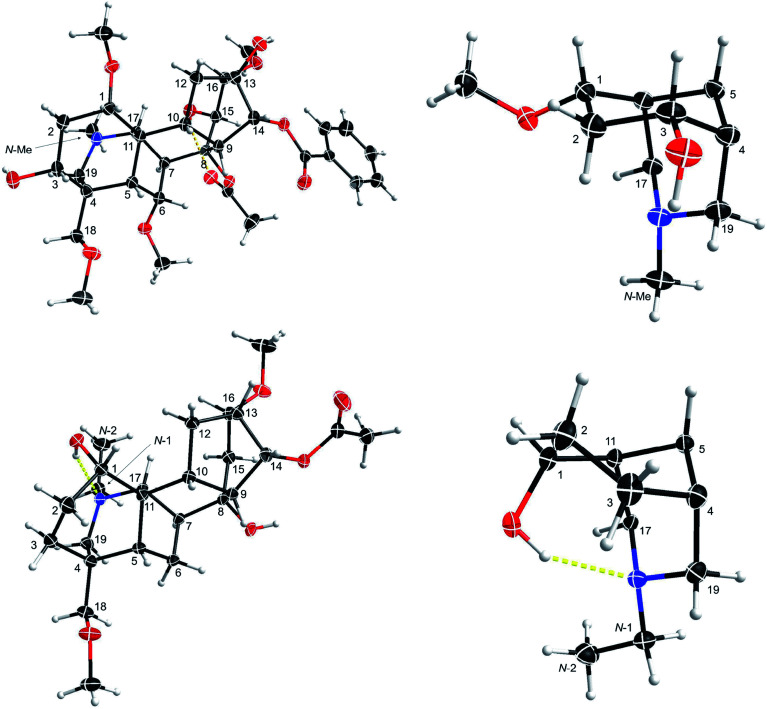
SXRD data of mesaconitine (5, upper) and condelphine (7, lower), and their A/E-[3.3.1]azabicyclic frames. ORTEP presentations of the crystal structures show the atom position with a 50% probability for each ellipsoid.

**Fig. 4:**
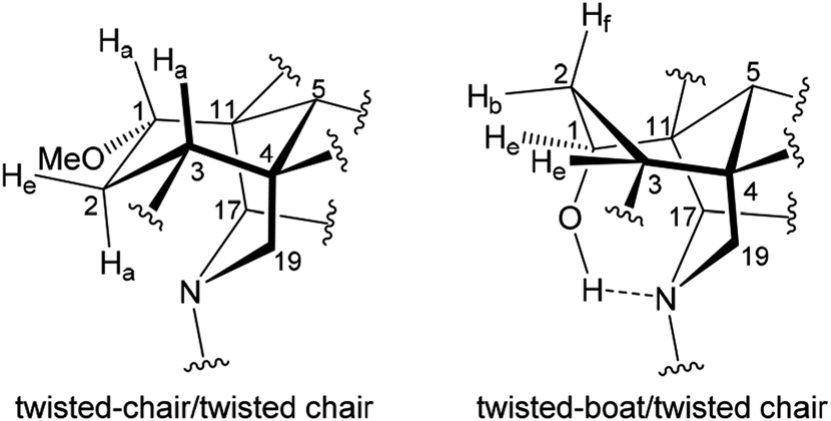
Conformations of A/E-[3.3.1]azabicycles of norditerpenoid alkaloids.

**Fig. 5:**
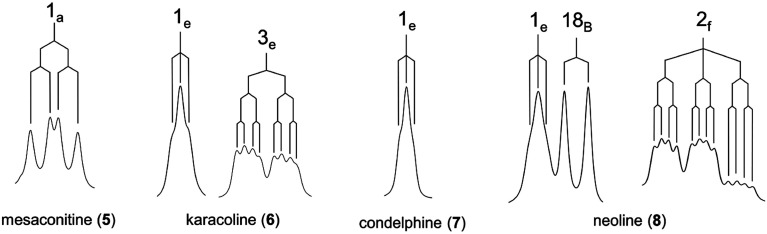
Coupling patterns of key ^1^H NMR signals of the selected norditerpenoid alkaloids.

Mesaconitine (5) with 1α-OMe, having an A-ring in the chair conformation proven by NOESY correlation 2-H_a_/19-H_e_ (Fig. 2) and SXRD data (Fig. 3, torsion angle *θ*_C1–C11–C4–C3_ = 9.28° > 4°, details of SXRD data are reported in Tables S5–S6†). It is highlighted that *δ* (2-H_a_) = 2.31 ppm is unusually larger than *δ* (2-H_a_) = 2.14 ppm by 0.17 ppm. The chemical shift of the axial proton of cyclohexane is normally larger than that of the geminal equatorial proton by ∼0.5 ppm due to the magnetic anisotropic effect.^23,24^ As the lone-pair electrons of the tertiary amine *N*-atom sterically compress 2-H_a_ in the half-cage A/E-[3.3.1]azabicycle, 2-H_a_ is therefore deshielded and its chemical shift becomes larger than that of 2-H_e_.^25,26^ In detail, 1-H_a_ (Fig. 4) of mesaconitine (5) resonates as a dd peak (*δ* = 3.11 ppm, ^3^*J*_aa_ = 9.0 Hz, ^3^*J*_ae_ = 6.2 Hz, Fig. 5) confirms that the A-ring adopts a chair conformation,^22^ and the smaller ^3^*J*_aa_ = 9.0 Hz (normally found in the range 12–14 Hz) indicates that this chair conformation is twisted as explained by Eliel.^27,28^

The A-rings of the selected 1α-OH alkaloids, karacoline (6), condelphine (7), and neoline (8), adopt boat conformations stabilized by an H-bond between the tertiary amine and 1α-OH,^21,29,30^ and they are proven to be in a twisted-boat conformation. For instance, the A-ring of neoline (8) adopts a boat conformation confirmed by NOESY correlation 2-H_f_/5-H, and 1-H_e_ resonates as a narrow t peak (*δ* = 3.66 ppm, ^3^*J*_be_ = ^3^*J*_ef_ = 3.3 Hz, proton labelling is shown in Fig. 4). It is notable that ^3^*J*_ef_ = 3.3 Hz of this 1-H_e_ of neoline (8) is different from ^3^*J*_ef_ = 5.3 Hz of 3-H_e_ that displays in the signal of 2-H_f_ (*δ* = 1.49 ppm, tdd, ^2^*J*_gem_ = ^3^*J*_af_ = 14.0 Hz, ^3^*J*_ef_ = 5.3 Hz, ^3^*J*_ef_ = 3.3 Hz) determining that the boat-conformer A-ring of neoline (8) is twisted, as ^3^*J*_ef_ = 5.3 Hz (3-H_e_) > ^3^*J*_ef_ = 3.3 Hz (1-H_e_) indicates dihedral angle ∠(1-H_e_)–C1–C2–(2-H_f_) < ∠(2-H_f_)–C2–C3–(3-H_e_) according to the Karplus relationship.^31^

This result reveals that the through-space repulsion between 1-H_e_ and 12-H_e′_ is twisting the A-ring in a boat conformation.^21,22^ Similar broad triplets of 1-H_e_ with small ^3^*J*_ef_ ∼ 3 Hz were observed in ^1^H NMR spectra of karacoline (6) (*δ* = 3.71 ppm, ^3^*J*_be_ = ^3^*J*_ef_ = 3.0 Hz) and condelphine (7) (*δ* = 3.73 ppm, ^3^*J*_be_ = ^3^*J*_ef_ = 3.0 Hz). In addition, 3-H_e_ of karacoline (6) resonates as a ddd peak (*δ* = 1.48 ppm, ^2^*J*_gem_ = 13.5 Hz, ^3^*J*_ef_ = 5.8 Hz, ^3^*J*_be_ = 3.0 Hz). Its ^3^*J*_ef_ (5.8 Hz) of 3-H_e_ is significantly different from the ^3^*J*_ef_ (3.0 Hz) of 1-H_e_ which supports the conclusion that the boat-like A-rings of these 1α-OH alkaloids are twisted. This conclusion that the A-rings of 1α-OH norditerpenoid alkaloids adopt twisted-boat conformations is supported by SXRD data of condelphine (7) free base (Fig. 3), in which the A-ring clearly adopts a twisted-boat conformer (torsion angle *θ*_C1–C11–C4–C3_ = 6.81° > 4°), and is stabilized by an H-bond between 1-OH and the tertiary N-atom.

As there is proximity between 6α-H_e′_ (or 6α-OMe) and 19-H_a_, *e.g.* in the NOESY correlation 6α-H_e′_/19-H_a_ of condelphine (7) (Fig. 2), the N-substituted piperidine E-rings of norditerpenoid alkaloids always adopt twisted-chair conformations.^21,22^ This conclusion of the E-ring adopting twisted-chair conformations is supported by the SXRD data of both mesaconitine (5) and condelphine (7) (torsion angles *θ*_C19–C4–C11–C17_ are 13.60° and 11.32°, respectively, >4°). Thus, the conformations of the A/E-[3.3.1]azabicycles of norditerpenoid alkaloids bearing 1α-OR are always twisted (Fig. 4).

**Fig. 6:**
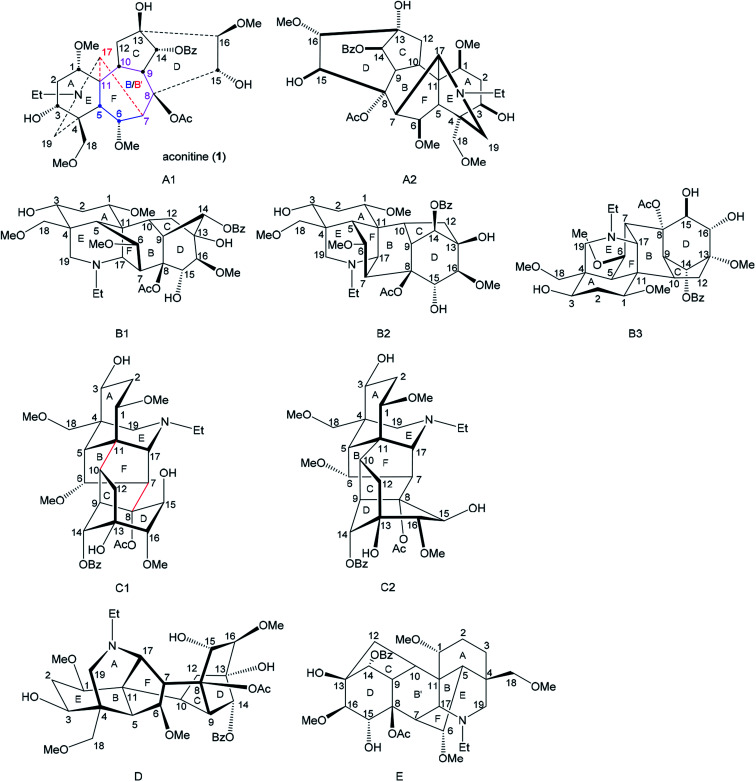
Aconitine (1) free base shown in different literature depictions.

For any research purpose in studies of norditerpenoid alkaloids, certain depictions must be employed to present structures of these polycyclic natural products, and a suitable depiction displaying favourable stereochemical information helps to achieve those specific goals. Such an understanding may possibly inspire research in various related areas, *e.g.* phytochemistry, organic synthesis, and structure–activity relationship (SAR) studies of biological activity. Several different reported depictions of norditerpenoid alkaloids are shown (Fig. 6) using aconitine (1) free base as an example.

The depiction A1 is currently the most popular reported in the field of phytochemistry.^32–36^ The depiction clearly displays three rings, A-, B- and C-ring, in suitable shapes. However, the conformations of some rings, *e.g.* A-, B-, and D-ring, are difficult to determine from this depiction. Through-space distances between some atoms are not favoured, *e.g.* C15 and C17 are actually close in space, but they are shown away in depiction A1.

Also, some bonds have to be depicted with a particularly long length, *e.g.* C7–C17. In some reported studies, especially in crystallographic studies,^9,37,38^ the 7-membered B-ring (C5–C11) refers to a 6-membered ring (B′-ring = C7–C8–C9–C10–C11–C17). Depiction A2 which is viewing depiction A1 from the other face (it is not the enantiomer) was also employed in order to present the D-, E- or F-ring on the β-face that might be beneficial for retrosynthetic analysis.^39–41^

Depictions B1 and B2 are also popularly used.^42,43^ Generally, these depictions B1 and B2 allow almost all the rings to be shown in the correct conformation, with well-balanced bond lengths (not of extreme length), and reasonable orientations of substituted functional groups. Specifically, depiction B1 shows the D-ring in the correct mono-flattened conformation. Depiction B2 allows space for the highly bridged B-ring, which brings benefit to any essential expansion of functional groups attached to the B-ring. However, depiction B2 cannot show the conformation of the D-ring, and therefore the orientations of the atoms/functional groups attached to this ring are ambiguously presented. Depictions B1 and B2 are widely employed by organic synthetic chemists for various reasons, *e.g.* B-ring with the largest number of bridgeheads displayed in the front may benefit retrosynthetic analysis,^44,45^ and it is also useful for designing disconnections of C-, D- and F-rings as these drawings correctly express the conformations.^46,47^ A rotated depiction of B2 (depiction B3, Fig. 2) was used for designing synthetic approaches starting with analogues of C/D-rings.^48^ Apart from the popular drawings A and B, an uncommon depiction C1 was also used,^49,50^ in which the relationship between the A/E/F-tricycle and C/D-bicycle are distinctly expressed, and these two cycles are clearly linked by bond C7–C8 and bond C10–C11 perhaps motivating retrosynthetic analysis. As the D-ring adopts a mono-flattened boat conformation with C14 at the flap, thus depiction C1 is slightly revised to C2.

It is notable that only the A-ring has 3 flexible C-atoms (C1–C3) that allow the conformation of the A-ring to flip; all other rings only have one or two flexible atoms in their skeleta. Therefore, conformations of these rings are inflexible, and the D-ring thereby adopts a fixed mono-flattened boat conformation. Furthermore, rare depictions D and E were also reported in older studies. Depiction D presents similar ideals to those of depiction C1,^51^ and depiction E was also shown in some crystallographic studies displaying piperidine E-, B′-, and D-rings in essentially ideal shapes.^9,20,37^

**Fig. 7:**
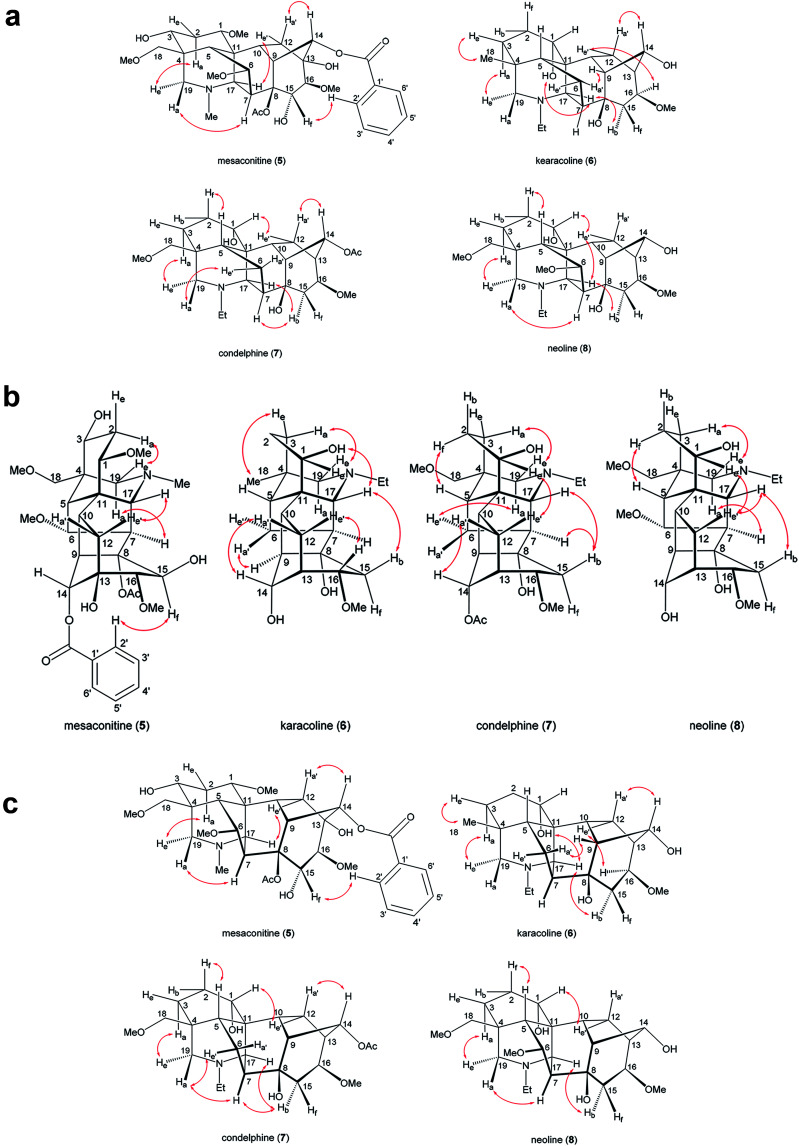
(a). Key NOESY correlations of the selected norditerpenoid alkaloids in depiction B1 (B1′). Depiction B1 shows the A-ring in chair conformation and depiction B1′ shows the A-ring in boat conformation. (b) Key NOESY correlations of the selected norditerpenoid alkaloids in depiction C2 (C2′). Depiction C2 shows the A-ring in chair conformation and depiction C2′ shows the A-ring in boat conformation. (c). Key NOESY correlations of the selected norditerpenoid alkaloids in depiction B4 (B4′). Depiction B4 shows the A-ring in chair conformation and depiction B4′ shows the A-ring in boat conformation.

In order to compare these depictions of norditerpenoid alkaloid, key NOESY correlations of mesaconitine (5), karacoline (6), condelphine (7), and neoline (8) are shown in depiction B1 (B1′) and C2 (C2′) (Fig. 7a and b) in comparison with those in depiction A1 (Fig. 2).

The A-rings of karacoline (6), condelphine (7), and neoline (8) adopt boat conformation proven by NOESY correlation 2-H_f_/5-H; however, this evidence cannot be unambiguously presented by depiction A1 as the conformation of the A-ring is hard to display in this depiction (Fig. 2). Moreover, some atoms that are close in space are not able to be displayed in this depiction, *e.g.* 7-H/19-H_a_, 15-H_b_/17-H (both on the α-face).

**Fig. 8:**
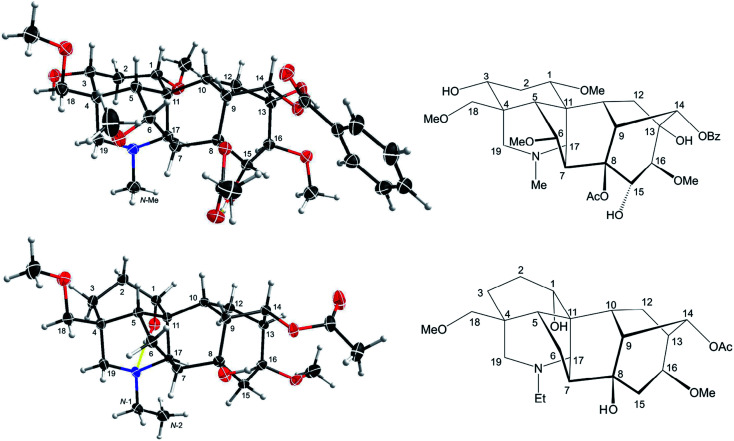
Depiction B4 (B4′) according to SXRD data of mesaconitine (5, upper) and condelphine (7, lower).

Depictions B1 (B1′) and C2 (C2′) indicate the conformations of the A-rings (Fig. 7a and b). However, some atoms that are close in space are separated from each other in depiction C2, *e.g.* 6-H_e′_/19-H_a_ of condelphine (7) and 15-H_b_/17-H of neoline (8). Besides, the internal space of depiction C2 is too narrow to allow the essential expansion of certain C–H bonds, *e.g.* protons attached to C1 and C12. Depiction B1 (B1′) almost ideally displays the distances between atoms in space except 7-H/19-H_a_, and the internal space of the B-ring of this depiction is narrow, in which some C–H bonds are hard to be shown clearly, *e.g.* protons attached to C6 and C9. According to the SXRD data of mesaconitine (5) and condelphine (7) (Fig. 8) and a report by Marion and co-workers,^43^ depiction B1 (B1′) is slightly modified to afford depiction B4 (B4′) (Fig. 7c), in which through-space distances between atoms are well-balanced and that aspect is favoured for the investigation of the stereochemistry of these norditerpenoid alkaloids.

### 2.1. Orientation of 7-H/OR

**Fig. 9:**
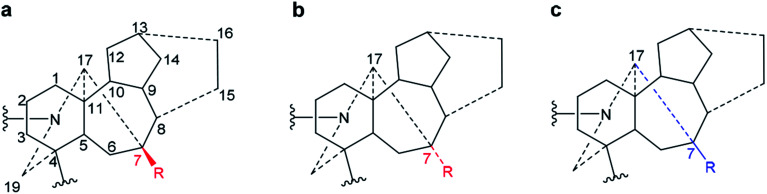
Skeleta of norditerpenoid alkaloids in depiction A1.

7-OR is the signature of the lycoctonine-subtype C_19_-diterpenoid alkaloids.^18^ In norditerpenoid alkaloids presented in depiction A1, 7-H/7-OR is popularly assigned as β-orientated (Fig. 9a).^32,33,36^ However, 7-H displayed in the SXRD data of mesaconitine (5) and condelphine (7) shown in an angle imitating depiction A1 do not show a typical β-orientation (Fig. 3). Moreover, NOESY correlations of 7-H/19-H_a_ of mesaconitine (5), condelphine (7), and neoline (8) were observed in this work, which proves that this 7-H cannot be β-orientated as typically drawn. In fact, a few uncommon assignments of 7α-OH of lycoctonine-type alkaloids have been reported (Fig. 9b),^37,52^ but they are not favoured as bond C7–C17 must be on the α-face. These 7-H/7-OR are equatorial β-orientated rather than typically axial β-orientated like protons or functional groups attached to C8 (Fig. 8), therefore, it is more appropriate and precise to draw the 7-H/7-OR in the plane of the paper if depiction A1 is employed for emphasizing this orientational feature of 7-H/7-OR, which has also been used in previous publications (Fig. 9c).^18,34,35^

## 3 Conclusions

A rare ^1^H NMR effect of steric compression is demonstrated in the A/E-[3.3.1]azabicycle of mesaconitine (b), in which the lone-pair electrons of the tertiary amine N-atom compress 2-H_a_ through space and therefore they deshield this proton. The A-rings of norditerpenoid alkaloids substituted at C1 with α-orientated oxygenated functional groups adopt twisted conformers due to the repulsion between atoms attached to C1 (H- or O-atoms in the equatorial position) and 12-H_e′_, no matter whether the A-rings are in chair or boat conformations. Different reported depictions of norditerpenoid alkaloids are compared according to the clarity of understanding for key NOESY correlations of the 4 selected alkaloid free bases, and the depiction B4 modified from depictions B1 and B2 is favoured to represent these natural products in publications as B4 expresses all the necessary information on conformation and orientation. 7-H/OR of norditerpenoid alkaloids is equatorially β-orientated, thus 7-H/7-OR is best drawn in the plane of the paper to distinguish its orientation from those typically axially β-orientated functional groups, *e.g.* 8-H/OR.

## Conflicts of interest

There are no conflicts to declare.

## Supplementary Material

RA-010-D0RA03811C-s001

RA-010-D0RA03811C-s002
