# Supplementary material for: The 1α-hydroxy-A-rings of norditerpenoid alkaloids are twisted-boat conformers
Source: RSC Adv. 2020 May 18;10(32):18797–805. doi: 10.1039/d0ra03811c (PMC9053968; doi:10.1039/d0ra03811c)
Supplement: RA-010-D0RA03811C-s001 [file RA-010-D0RA03811C-s001.pdf]

Electronic Supplementary Material (ESI) for RSC Advances.
This journal is © The Royal Society of Chemistry 2020

The 1α-Hydroxy-A-rings of Norditerpenoid Alkaloids are Twisted-Boat Conformers

Electronic Supplementary Information

Ziyu Zeng,[a] Ashraf M. A. Qasem,[a] Gabriele Kociok-Köhn,[b] Michael G. Rowan,[a] and Ian S. Blagbrough*[a]

[a]Department of Pharmacy and Pharmacology, [b]Material and Chemical Characterisation (MC^2), University of Bath, Bath BA2 7AY, U.K.

Table of Contents

Complete NMR assignments and revision of ^{13}C NMR assignments 2
Recrystallization and data of single-crystal X-ray determinations 6
NMR spectra 8
References 21

General methods

Chemicals and materials

Karacoline was purchased from Latoxan (France). Condelphine was donated by Carbosynth (UK). Neoline was purchased from Carbosynth (UK). *d*-Chloroform was used for NMR experiments (99.8% D atom, Cambridge Isotope Laboratories, Inc., USA). Mesaconitine was extracted and then purified by sulfuric acid acid-base cycling from the ground roots of *Aconitum napellus*. After column chromatography to homogeneity it was indistinguishable from a commercial sample (Sigma-Aldrich, UK).

Instrumentation

^{1}H NMR spectra were recorded on a Bruker Avance III spectrometers (^{1}H Larmor precession frequency 500 MHz) at 25 °C. Chemical shifts were expressed in parts per million (ppm) downfield shift from tetramethylsilane (TMS) or 3-(trimethylsilyl)-propionic-2,2,3,3-d_4 acid sodium salt (TMSP) as internal or external standards, and residual (protio) solvent peaks were also used as internal standards if required. Chemical shifts (δ_H) were reported as position (accurate δ_H of overlapping signals were extracted from 2D NMR spectra, e.g. HSQC, COSY, and NOESY), relative integral, multiplicity and assignment. Multiplicity was abbreviated: s = singlet, d = doublet, t = triplet, q = quartet, quin = quintet, m = multiplet; br = broad. Coupling constants (*J*) are line separations (absolute values expressed in Hertz, Hz), rounded and rationalised to 0.1 Hz.

^{13}C NMR spectra were recorded with complete proton decoupling on Bruker Avance III spectrometers (^{13}C Larmor precession frequency 125 MHz) at 25 °C as well as 2D NMR experiments including HSQC, H2BC, and HMBC. Chemical shifts are expressed in ppm downfield shift from TMS or TMSP as internal or external standards, and solvent peaks were also used as internal standards if required, and they were reported as position (δ_C), number of attached proton(s) (CH_3, CH_2, CH, quat = quaternary) and assignment.

Intensity data of single-crystal X-ray crystallography were collected at 150±2 K on a Rigaku SuperNova, EosS2 single crystal diffractometer using monochromated Cu-Kα radiation (λ = 1.54184 Å) which confirmed the absolute structure of the studied materials.

Complete NMR assignments and revision of ^{13}C NMR assignments

Table S1. NMR data of mesaconitine [**5**, $CDCl_3$; ^{1}H 500 MHz; ^{13}C 125 MHz; both were calibrated with residual $CHCl_3$ (7.26 ppm)/$CDCl_3$ (77.16 ppm)]

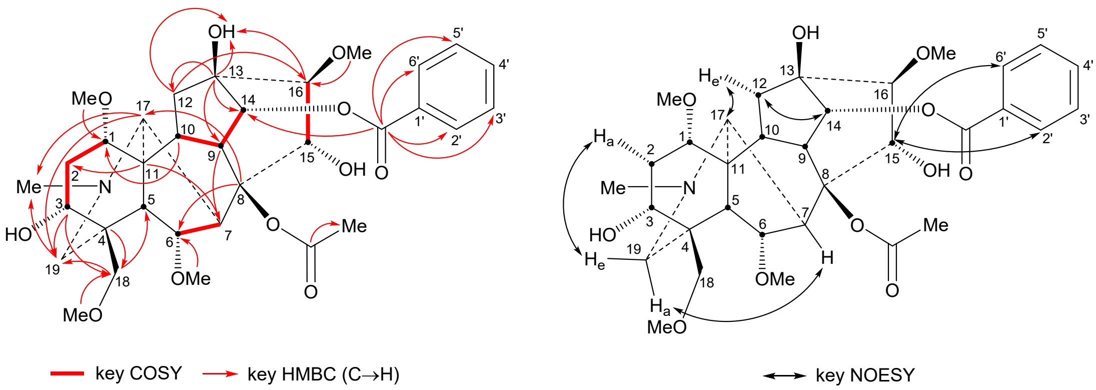

Carbon	δ_C	δ_H, multiplicity (*J*, in Hz; orientation or label[a])	key HMBC (C→H)	key NOESY
1	82.57[b]	3.11, dd (9.0, 6.2)	-	-
2	34.24[b]	2.31, m (α/a)[c]	-	19-H$_e$
		2.14, m (β/e)[c]		-

3	71.35	3.74, m[b]	19-H_a, 19-H_e, 18-H_A, 18-H_B	-
4	43.80[b]	-	18-H_B	-
5	46.77[b]	2.06, d (6.7)	-	-
6	83.31[b]	4.03, d (6.7)	-	-
7	43.60[b]	2.88, br s	-	19-H_a
8	91.99	-	6-H, 17-H	-
9	44.38[b]	2.90, dd (7.3, 5.1)	7-H	-
10	40.97	2.10, m[c]	1-H	-
11	50.08	-	2-H_e, 7-H	-
12	35.93[b]	2.83, d (10.0; α/e')<br>2.11, m (β/a')[c]	13-OH, 16-H	17-H<br>14-H
13	74.18	-	9-H, 12-$H_{a'}$, 12-$H_{e'}$, 14-H, 13-OH	-
14	78.99[d]	4.86, t (5.1)	-	12-$H_{a'}$
15	78.99[d]	4.46, dd (5.3, 2.8)[e]	-	2'-H, 6'-H
16	90.15	3.32, d (5.3)	13-OH	-
17	62.30	3.05, br s	19-H_e, *N*Me	12-$H_{e'}$
18	76.50	3.64, d (9.0; H_B)<br>3.55, d (9.0; H_A)	*N*Me	-<br>-
19	49.59	2.77, d (11.4; e)<br>2.36, m (a)[c]	5-H, 17-H, *N*-1_A, *N*-1_B	2-H_a<br>7-H
*N*Me	42.57	2.34, s	-	-
**CO**Me	172.58	-	CO**Me**	-
CO**Me**	21.58	1.37	-	-
**CO**Ph	166.23	-	14-H, 2'-H, 3'-H, 5'-H, 6'-H	-
1'	129.90	-	-	-
2', 6'	129.75	8.02, dd (7.6, 1.3)	-	15-H
3', 5'	128.80	7.45, d (7.6)	-	-
4'	133.45	7.57, tt (7.6, 1.3)	-	-
1-OMe	56.51	3.28, s	1-H	-
6-OMe	58.09	3.16, s	6-H	-
16-OMe	61.22	3.73, s	16-H	-
18-OMe	59.27	3.29 ,s	18-H_A, 18-H_B	-
13-OH	-	3.92, s	-	-
15-OH	-	4.35, d (2.8)[e]	-	-

[a] Orientation label: a = axial, e = equatorial, b = bowsprit, f = flagpole, a' = pseudo-axial, e' = pseudo-equatorial.
[b] New assignment.[1,2]
[c] The accurate δ_H of the overlapping signal was extracted from HSQC.
[d] C14 and C15 are fully overlapped.
[e] 15-H COSY correlates with 15-OH.

Table S2. NMR data of karacoline (**6**, $CDCl_3$; ^{1}H 500 MHz; ^{13}C 125 MHz; both were calibrated with TMS)

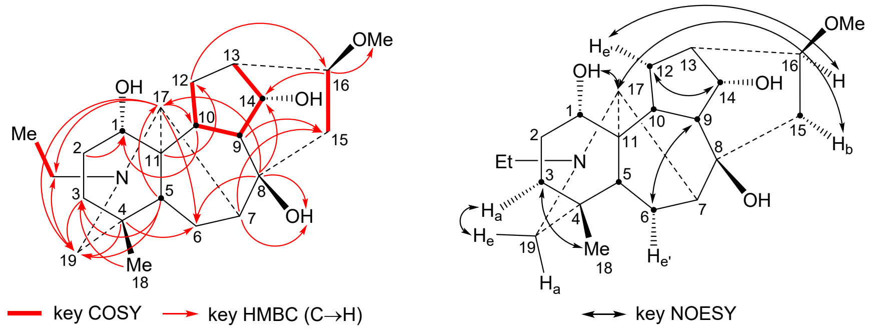

Carbon	δ_C[a]	δ_H, multiplicity (*J*, in Hz; orientation or label[b])	key HMBC (C→ H)	key NOESY
1	72.53	3.71, br t (3.0)	-	-
2	29.74	1.62, m (2-H_B)[c,d,e]<br>1.58, m (2-H_A)[c,d,f]	1-H	-<br>-
3	31.34	1.74, td (13.5, 5.8; α/a)<br>1.48, ddd (13.5, 5.8, 3.0; β/e)	19-H_e	19-H_e<br>18-H
4	32.92	-	19-H_e, 6-$H_{a'}$	-

5	46.63	1.61, m[c]	3-H_a, 17-H, 19-H_e	-
6	25.14	1.92, dd (14.9, 7.2; α/a')	-	9-H
		1.58, m (α/e')[c,f]		-
7	45.08	2.06, m[c,g]	8-OH, 15-H_b	-
8	74.17	-	8-OH, 15-H_b ,17-H	-
9	46.74	2.23, t (5.9)	14-H, 17-H	6-$H_{a'}$
10	44.06	1.83, dt (12.0, 5.9)	1-H	-
11	48.81	-	12-$H_{e'}$	-
12	28.21	2.03, m (β/a')[c]	16-H	14-H
		1.62, m (α/e')[c,e]		16-H
13	39.80	2.34, dd (7.3, 5.9)	-	-
14	75.96	4.23, br dd (7.3, 5.9)	-	12-$H_{a'}$
15	42.28	2.41, dd (15.6, 9.2; α/b)	-	17-H
		2.06, m (β/f)[c,g]		-
16	81.85	3.38, br dd (9.2, 5.9)[h]	16-OMe	12-$H_{e'}$
17	63.43	2.78, br s	6-$H_{a'}$, 10-H, 19-H_e, *N*-1_B	1-OH, 15-H_b
18	27.58	0.88, s	3-H_a,	3-H_e
19	60.26	2.28, d (e)	*N*-1_B	3-H_a
		2.07, m (a)[c]		-
*N***CH₂**CH₃ (*N*-1)	48.42	2.53, dt (14.4, 7.2)	-	-
		2.44, m[c]		
*N*CH₂**CH₃** (*N*-2)	13.14	1.11, t (7.2)	-	-
16-OMe	56.34	3.35, s	-	-
1-OH	-	6.96, br s	-	17-H
8-OH	-	3.03, s	-	-
14-OH	-	3.38, m[h]	-	-

[a] These ^{13}C NMR assignments are supported by a reported work.[3]
[b] Orientation label: a = axial, e = equatorial, b = bowsprit, f = flagpole, a' = pseudo-axial, e' = pseudo-equatorial.
[c] The accurate δ_H of the overlapping signal was extracted from HSQC.
[d] No evidence was obtained to assign the orientations of protons attach C2.
[e] These ^{1}H NMR signals are fully overlapped.
[f] These ^{1}H NMR signals are fully overlapped.
[g] These ^{1}H NMR signals are fully overlapped.
[h] ^{1}H NMR signal of 16-H resonating at 3.38 ppm showed an integral of 2H, thus ^{1}H NMR signal 14-OH is underneath of 16-H, and this assignment of 14-OH was further confirmed by a H2BC correlation between 14-OH/C14.

Table S3. NMR data of condelphine [**7**, $CDCl_3$; ^{1}H 500 MHz; ^{13}C 125 MHz; both were calibrated with residual $CHCl_3$ (7.26 ppm)/$CDCl_3$ (77.16 ppm)]

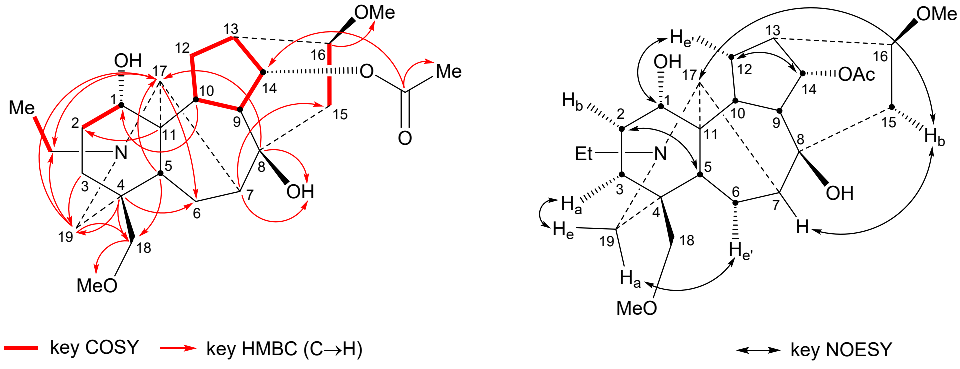

Carbon	δ_C	δ_H, multiplicity (*J*, in Hz; orientation or label[a])	key HMBC (C→H)	key NOESY
1	72.21	3.73, br t (3.0)	-	12-$H_{e'}$
2	29.85[b,c]	1.60, m (α/b)[d]	-	-
		1.56, m (β/f)[d]		5-H
3	26.72[b,c]	1.87, m (β/e)[d]	19-H_e	-
		1.63, m (α/a)[d]		19-H_e
4	37.31[b,c]	-	19-H_a, 19-H_e, 18-H_A, 18-H_B	-

5	41.49	1.84, br s	17-H, 18-H$_A$, 18-H$_B$	2-H$_f$
6	25.16	1.82, m (α/a')[d]<br>1.66, m (α/e')[d]	-	-<br>19-H$_a$
7	45.59	2.07, m[c]	8-OH, 15-H$_b$	15-H$_b$
8	74.92	-	8-OH, 17-H	-
9	44.91	2.26, m[d]	-	-
10	43.39[b]	1.90, m[d,e]	1-H	-
11	49.03	-	2-H$_b$	-
12	29.23[b,c]	2.09, m (β/a')[d]<br>1.70, m (α/e')[d]	-	14-H<br>1-H
13	36.68[b]	2.63, dd (7.8, 5.0)	-	-
14	77.27	4.86, d (5.0)	-	12-H$_{a'}$
15	42.78	2.30, m (α/b)[d]<br>1.90, m (β/f)[d,e]	-	7-H, 17-H
16	82.17	3.28, m[b]	16-OMe	-
17	63.82	2.73, br s	6-H, *N*-1$_A$, *N*-1$_B$	15-H$_b$
18	79.10	3.15, d (8.8; H$_B$)<br>3.00, d (8.8; H$_A$)	18-OMe	-<br>-
19	56.68	2.32, m (e)[d]<br>2.05, m (a)[d]	17-H, 18-H$_A$, 18-H$_B$, *N*-1$_A$, *N*-1$_B$	3-H$_a$<br>-
*N***CH$_2$**CH$_3$ (*N*-1)	48.62	2.53, dt (19.4, 7.2; *N*-1$_B$)<br>2.44, dt (19.4, 7.2; *N*-1$_A$)	-	-
*N*CH$_2$**CH$_3$** (*N*-2)	13.18	1.11, t (7.2)	-	
**CO**Me	170.61	-	CO**Me**	-
CO**Me**	21.49	2.06	-	-
16-OMe	56.21	3.26, s	-	-
18-OMe	59.60	3.32 ,s	-	-
1-OH	-	7.12, br s	-	-
8-OH	-	2.14, s	-	-

[a] Orientation label: a = axial, e = equatorial, b = bowsprit, f = flagpole, a' = pseudo-axial, e' = pseudo-equatorial.
[b] New assignment.[1]
[c] New assignment.[4]
[d] The accurate δ$_H$ of the overlapping signal was extracted from HSQC.
[e] 10-H and 15-H$_b$ are full overlapped.

Table S4. NMR data of neoline (**8**, CDCl$_3$; ^{1}H 500 MHz; ^{13}C 125 MHz; both were calibrated with TMS; ppm)

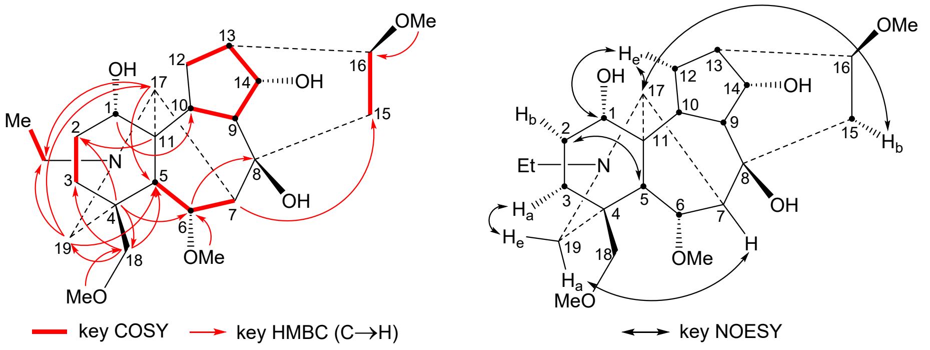

Carbon	δ$_C$[a]	δ$_H$, multiplicity (*J*, in Hz; orientation or label[b])	key HMBC (C→H)	key NOESY
1	72.23	3.66, br t (3.3)	10-H	12-H$_{e'}$
2	29.90	1.56, m (α/b)[c]<br>1.49, tdd (14.0, 5.3, 3.3; β/f)	-	5-H<br>-
3	29.39	1.90, m (β/e)[c]<br>1.62, td (14.0, 4.9; α/a)	-	19-H$_e$<br>-
4	38.13	-	6-H, 18-H$_A$, 18-H$_B$	-
5	44.88	2.17, m[c]	-	2-H$_b$
6	83.10	4.17, d (6.7)	8-H	-
7	52.15	2.00, br s	15-H$_f$, 15-H$_b$	19-H$_a$
8	74.21	-	-	-

9	48.32[d]	2.19, m[c]	-	-
10	44.13	1.86, m[c]	-	-
11	49.49	-	2-H_b	-
12	29.22	2.04, m (β/a')[c]	-	-
		1.72, dd (14.6, 5.1; α/e')		1-H, 17-H
13	40.27	2.29, dd (7.0, 5.0)	-	-
14	76.05	4.22, t (5.0)	-	-
15	42.87	2.38, dd (15.9, 9.2; α/b)	-	17-H
		2.06, td (15.9, 5.8; β/f)[c]		-
16	81.77	3.37, dd (9.2, 5.8)	-	-
17	63.82	2.68, br s	5-H, *N*-1_A, *N*-1_B,	12-$H_{e'}$, 15-H_b
18	80.27	3.64, d (8.1; H_B)	3-H_e, 5-H, 19-H_a	-
		3.26, d (8.1; H_A)		-
19	57.01	2.71, m (10.7; a)	5-H, 17-H, *N*-1_A, *N*-1_B,	7-H
		2.32, d (10.7; e)		3-H_e
*N***CH₂**CH₃ (*N*-1)	48.30[d]	2.57, dt (14.3, 7.2; *N*-1_B)	-	-
		2.50, dt (14.3, 7.2; *N*-1_A)		-
*N*CH₂**CH₃** (*N*-2)	13.07	1.13, t (7.2)	-	-
6-OMe	57.89	3.34, s[e]	6-H	-
16-OMe	56.32	3.34, s[e]	16-H	-
18-OMe	59.20	3.33, s	18-H_A, 18-H_B	-
OH	-	7.39, br s[a]	-	-
		3.07, br s[a]		
		2.91, br s[a]		

[a] These ^{13}C NMR assignments are supported by a reported study.[5]

[b] Orientation label: a = axial, e = equatorial, b = bowsprit, f = flagpole, a' = pseudo-axial, e' = pseudo-equatorial.

[c] The accurate δ_H of the overlapping signal was extracted from HSQC.

[d] These ^{13}C NMR signals were differentiated by DEPT.

[e] 6-OMe and 16-OMe are fully overlapped.

Recrystallization and data of single-crystal X-ray determinations

A mixture of DCM/EtOAc was added dropwise to a glass vial (5-10 mL) containing the indicated sample (~5 mg) heated on a water bath (40-60 °C) with slow shaking until the sample was fully dissolved, and then the glass vial was covered with foil, allowed to cool to 20 °C, and then to partially evaporate at 20 °C for the required period for growing crystals that are suitable for single crystal X-ray analysis. Mesaconitine (**5**): 3 day; condelphine (**7**): 4 days. Intensity data were collected at 150±2 K on a Rigaku SuperNova, EosS2 single crystal diffractometer using monochromated Cu-Kα radiation (λ = 1.54184 Å) which confirmed the absolute structure of the studied materials.

Unit cell determination, data collection and data reduction were performed using the CrysAlisPro software.[6] An empirical absorption correction using spherical harmonics and an additional Numerical absorption correction based on gaussian integration over a multifaceted crystal model was employed. The structures were solved with SHELXT[7] and refined by a full-matrix least-squares procedure based on F^2 (SHELXL-2018/3).[7] All non-hydrogen atoms were refined anisotropically. CH hydrogen atoms were placed onto calculated positions and refined using a riding model. All OH hydrogen atoms have been located in the difference Fourier map and were refined freely. Details of SXRD data are reported in Table S5-S6.

Table S5. Crystal data and structure refinement for mesaconitine (**5**) (**s20phar2**) (CCDC 1990874)

Identification code	s20phar2	
Empirical formula	C33 H45 N O11	
Formula weight	631.70	
Temperature	150.00(10) K	
Wavelength	1.54184 Å	
Crystal system	Orthorhombic	
Space group	$P2_12_12_1$	
Unit cell dimensions	a = 12.70194(13) Å	α= 90°.
	b = 15.37677(17) Å	β= 90°.
	c = 15.55588(17) Å	γ = 90°.

Volume	3038.29(6) Å^3
Z	4
Density (calculated)	1.381 Mg/m^3
Absorption coefficient	0.857 mm^{-1}
F(000)	1352
Crystal size	0.282 x 0.136 x 0.116 mm^3
Theta range for data collection	4.042 to 73.372°.
Index ranges	-15<=h<=15, -17<=k<=19, -19<=l<=19
Reflections collected	55167
Independent reflections	6062 [R(int) = 0.0347]
Completeness to theta = 67.684°	100.0 %
Absorption correction	Gaussian
Max. and min. transmission	1.000 and 0.748
Refinement method	Full-matrix least-squares on F^2
Data / restraints / parameters	6062 / 0 / 424
Goodness-of-fit on F^2	1.039
Final R indices [I>2sigma(I)]	R1 = 0.0268, wR2 = 0.0680
R indices (all data)	R1 = 0.0271, wR2 = 0.0683
Absolute structure parameter	0.03(4)
Extinction coefficient	n/a
Largest diff. peak and hole	0.182 and -0.168 e.Å^{-3}

Table S6. Crystal data and structure refinement for condelphine (**7**) (**s20phar1**) (CCDC 1990875)

Identification code	s20phar1	
Empirical formula	C25 H39 N O6	
Formula weight	449.57	
Temperature	150.00(10) K	
Wavelength	1.54184 Å	
Crystal system	Monoclinic	
Space group	P2$_1$	
Unit cell dimensions	a = 8.92530(9) Å	α= 90°.
	b = 13.08371(15) Å	β= 93.4553(9)°.
	c = 9.80866(10) Å	γ = 90°.
Volume	1143.33(2) Å^3	
Z	2	
Density (calculated)	1.306 Mg/m^3	
Absorption coefficient	0.747 mm^{-1}	
F(000)	488	
Crystal size	0.388 x 0.374 x 0.094 mm^3	
Theta range for data collection	4.516 to 72.905°.	
Index ranges	-11<=h<=11, -16<=k<=14, -12<=l<=12	
Reflections collected	18838	
Independent reflections	4338 [R(int) = 0.0245]	
Completeness to theta = 67.684°	100.0 %	
Absorption correction	Gaussian	
Max. and min. transmission	1.000 and 0.467	
Refinement method	Full-matrix least-squares on F^2	
Data / restraints / parameters	4338 / 1 / 301	
Goodness-of-fit on F^2	1.062	
Final R indices [I>2sigma(I)]	R1 = 0.0299, wR2 = 0.0757	
R indices (all data)	R1 = 0.0301, wR2 = 0.0759	
Absolute structure parameter	-0.02(5)	
Extinction coefficient	n/a	
Largest diff. peak and hole	0.184 and -0.220 e.Å^{-3}	

NMR spectra

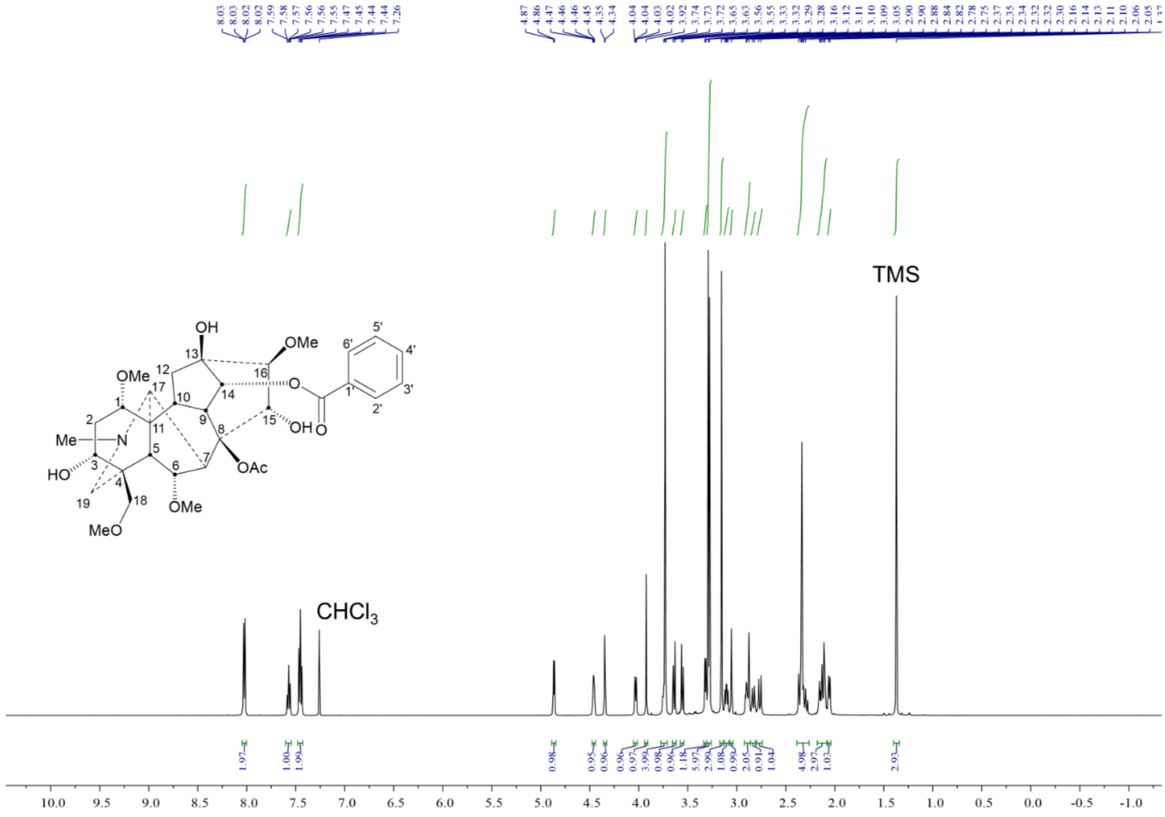

Figure S1. ^{1}H NMR spectrum of mesaconitine (**5**)

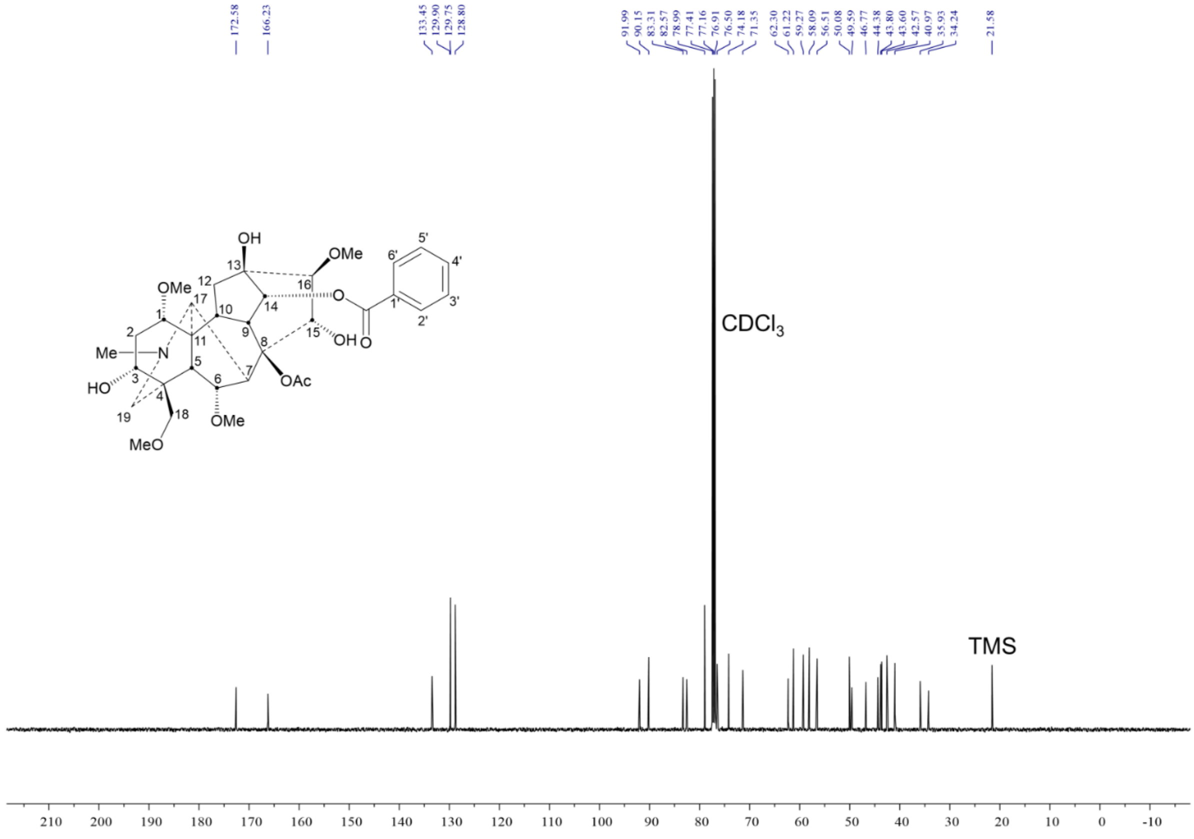

Figure S2. ^{13}C NMR spectrum of mesaconitine (**5**)

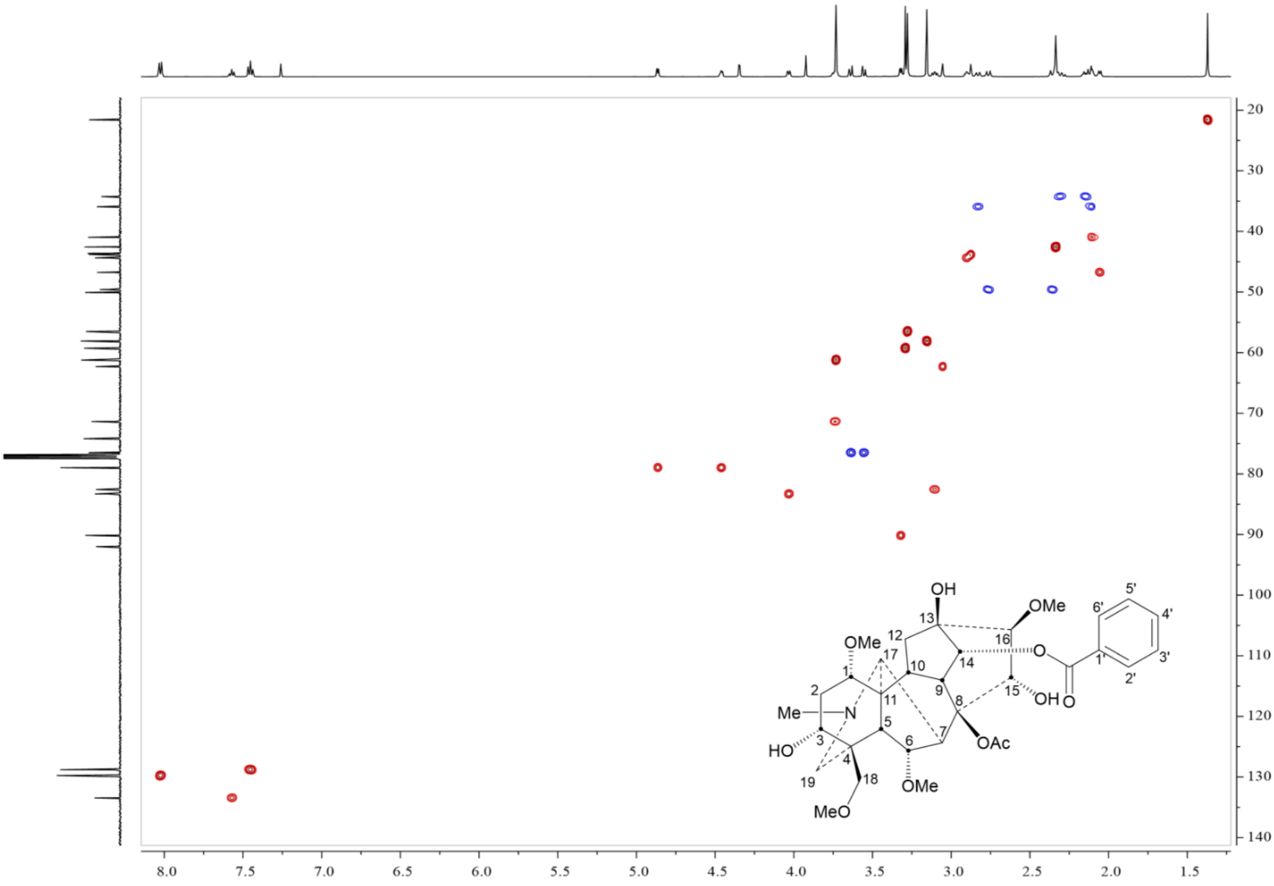

Figure S3. HSQC spectrum of mesaconitine (**5**)

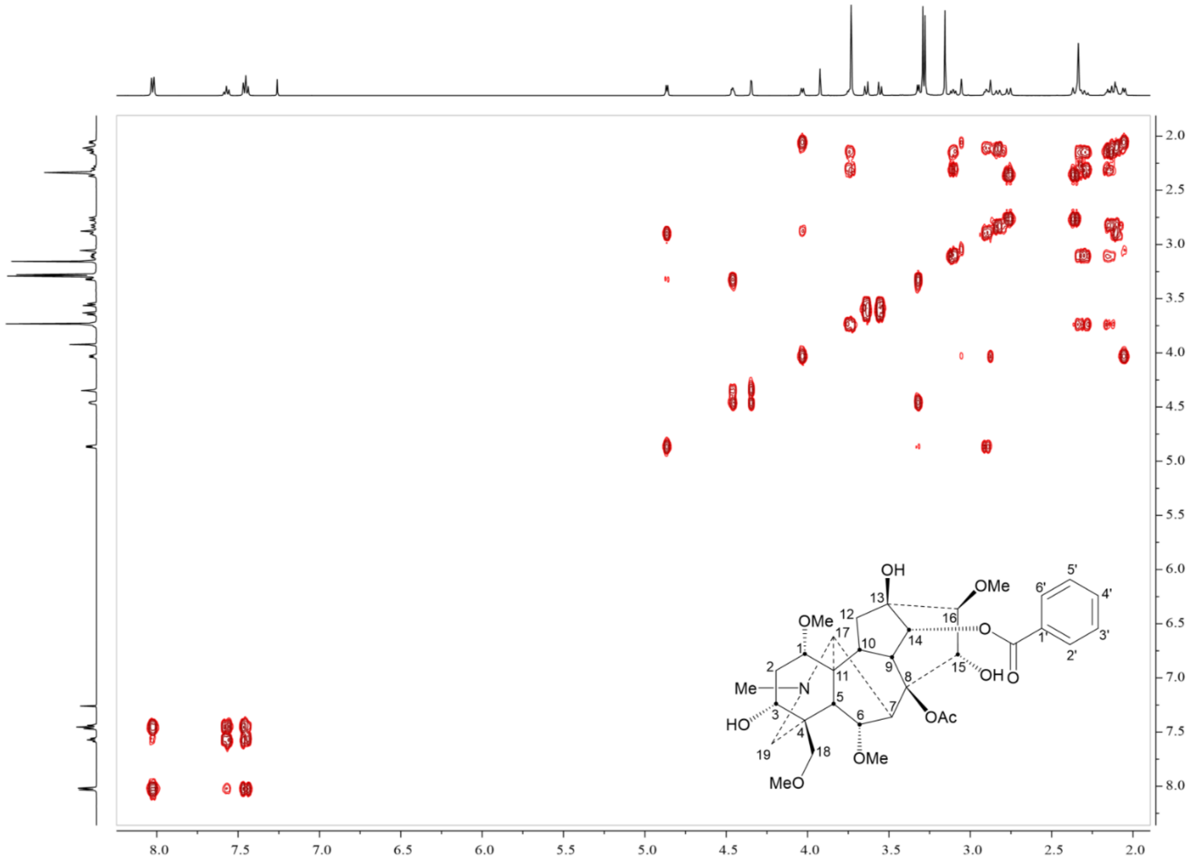

Figure S4. COSY spectrum of mesaconitine (**5**)

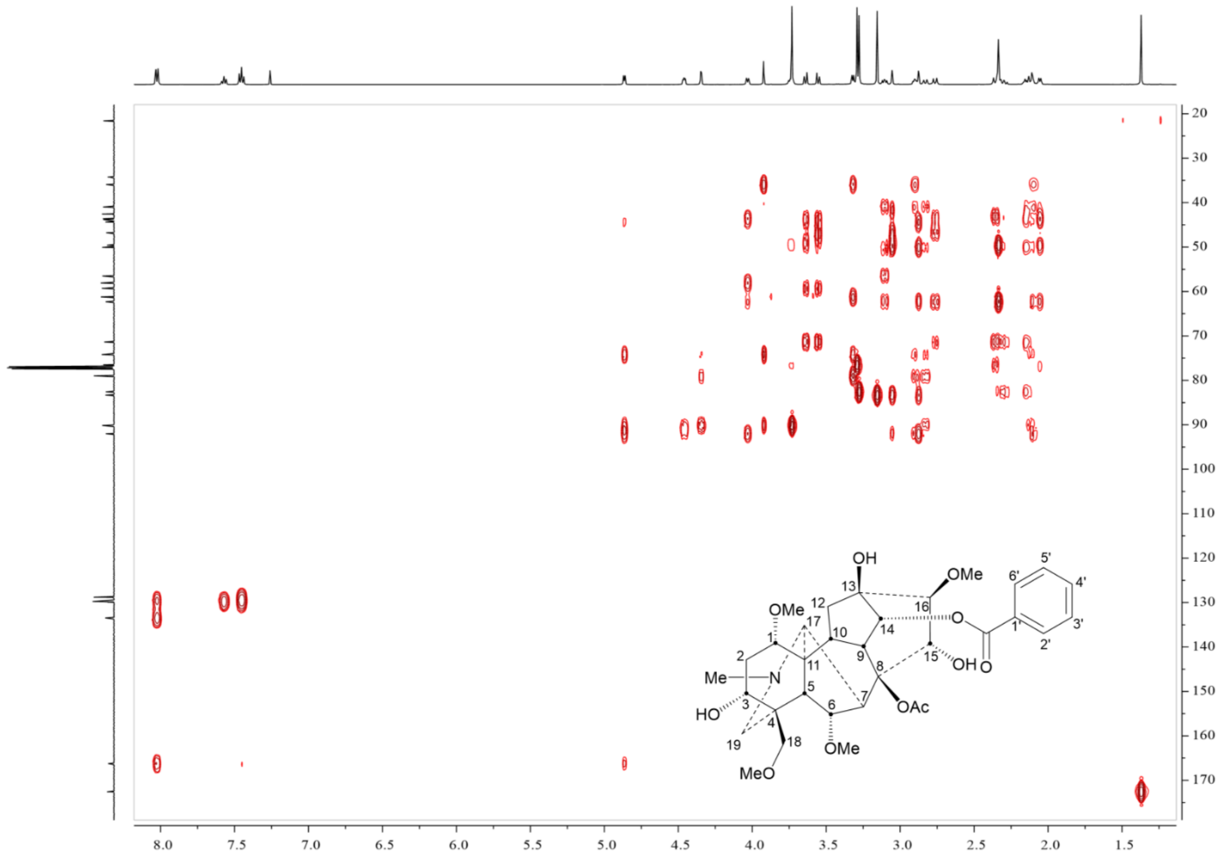

Figure S5. HMBC spectrum of mesaconitine (**5**)

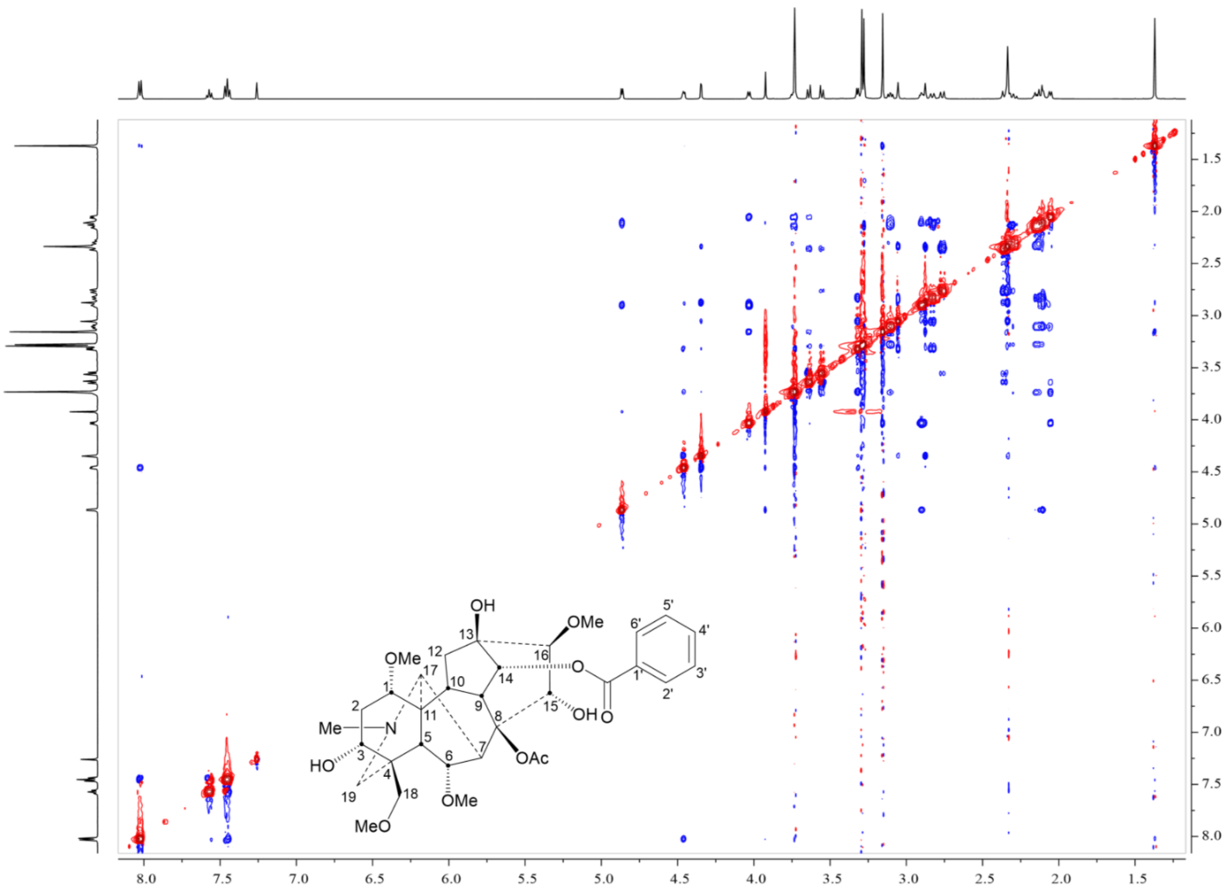

Figure S6. NOESY spectrum of mesaconitine (**5**)

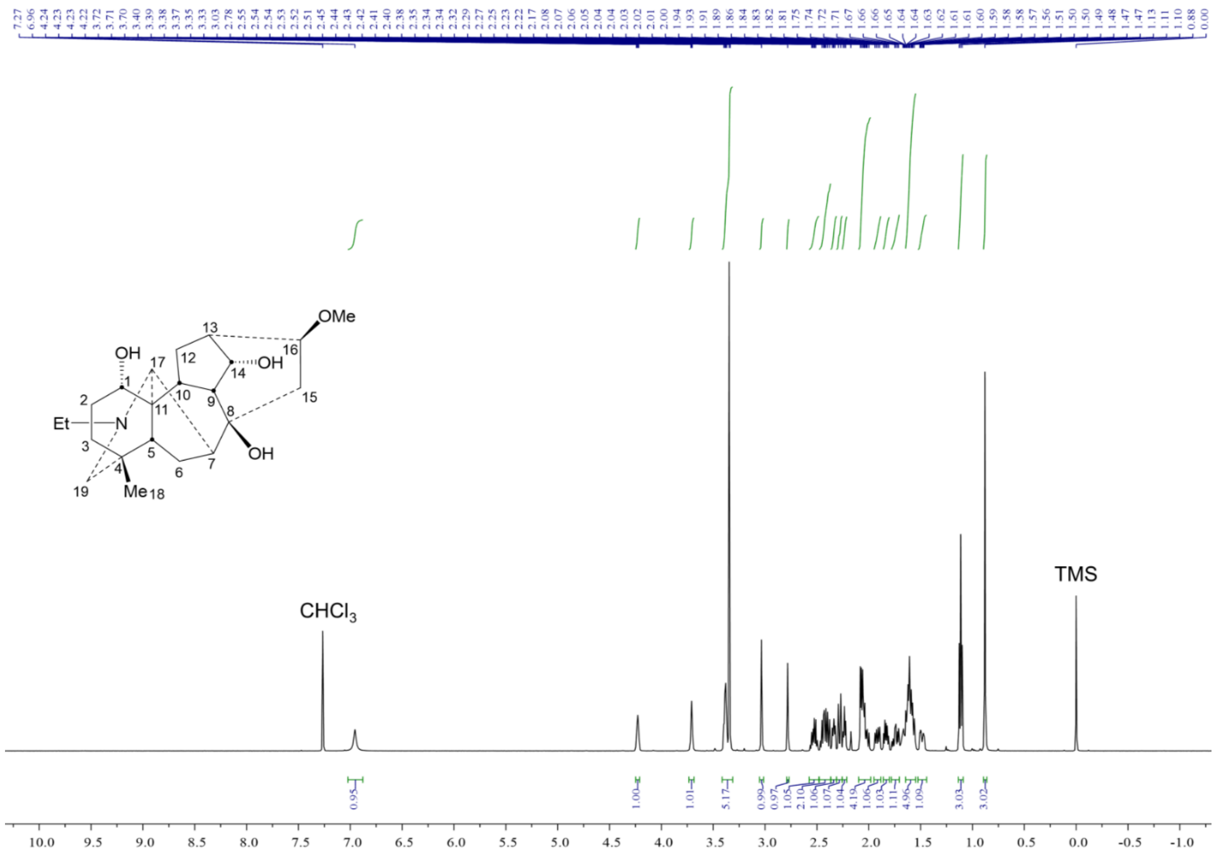

Figure S7. ^{1}H NMR spectrum of karacoline (**6**)

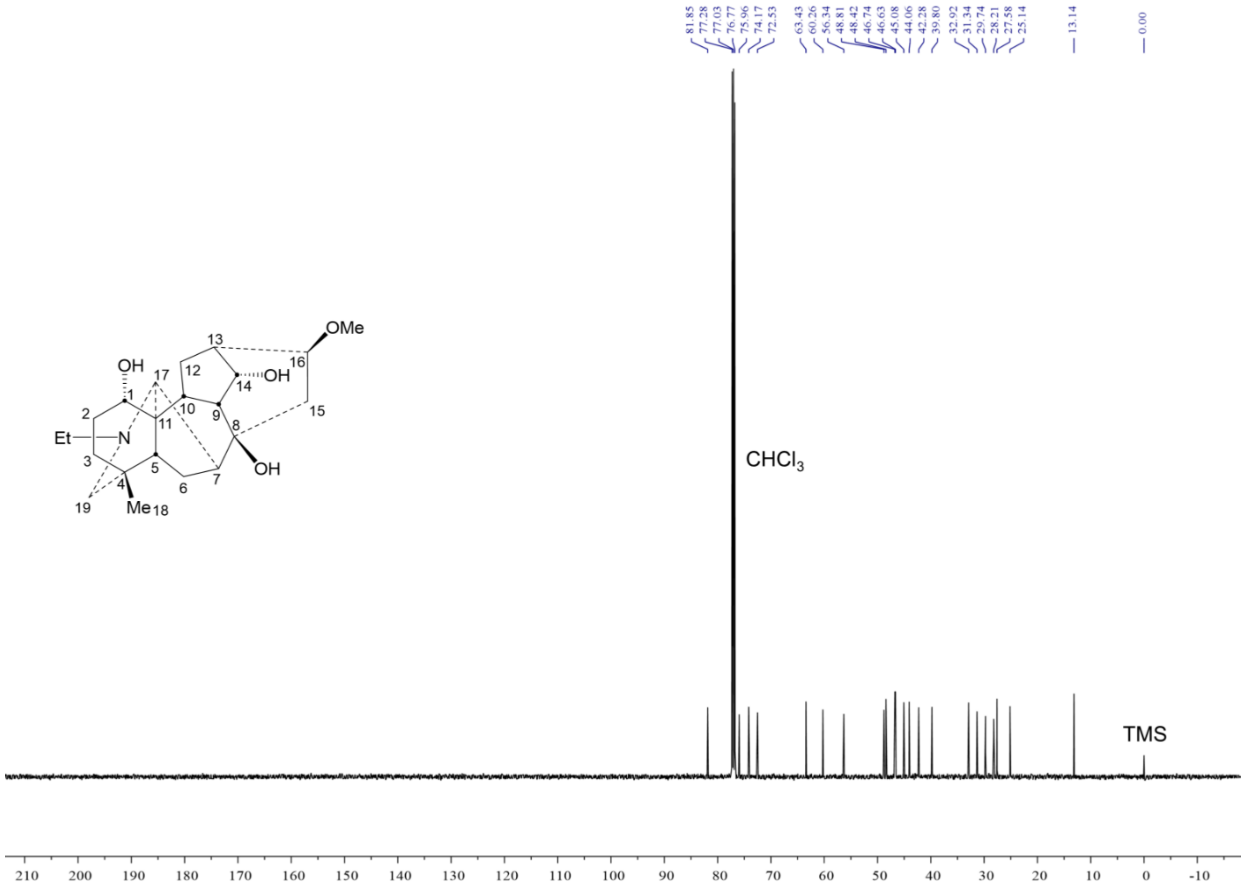

Figure S8. ^{13}C NMR spectrum of karacoline (**6**)

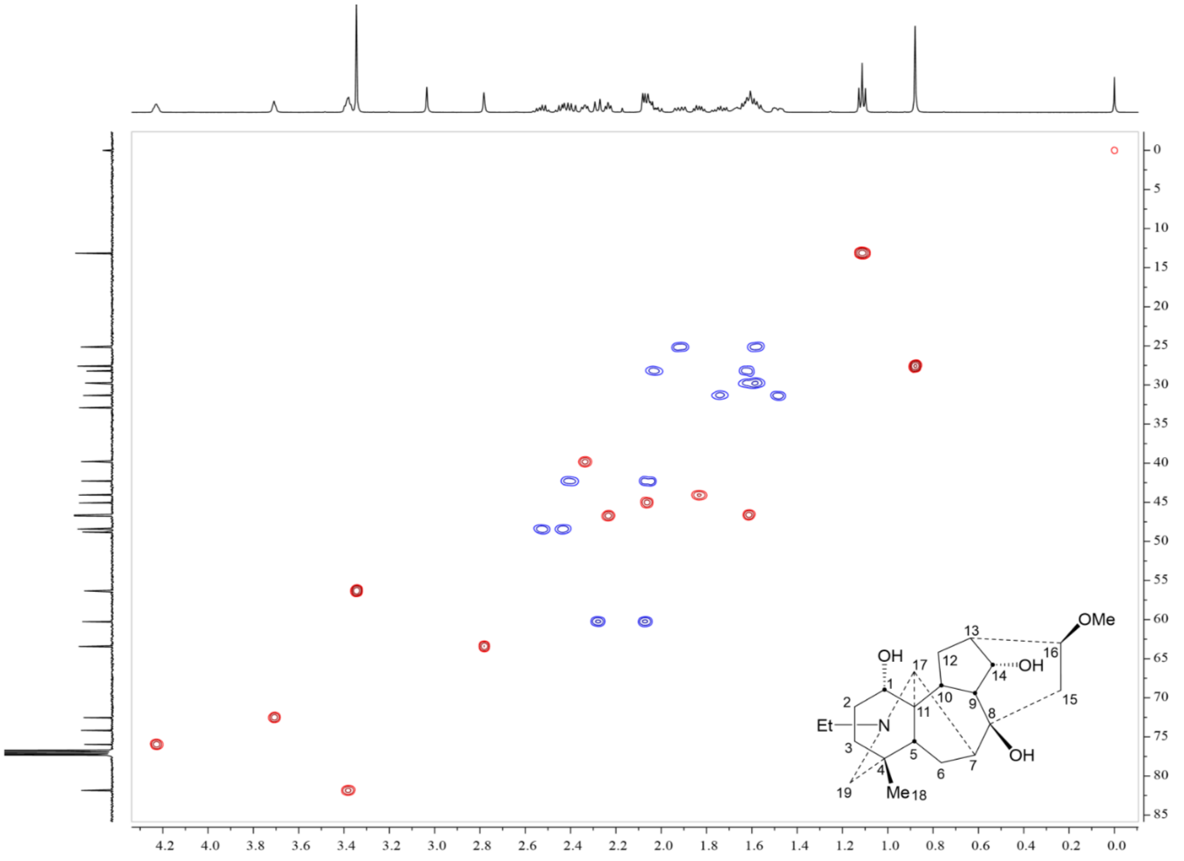

Figure S9. HSQC spectrum of karacoline (**6**)

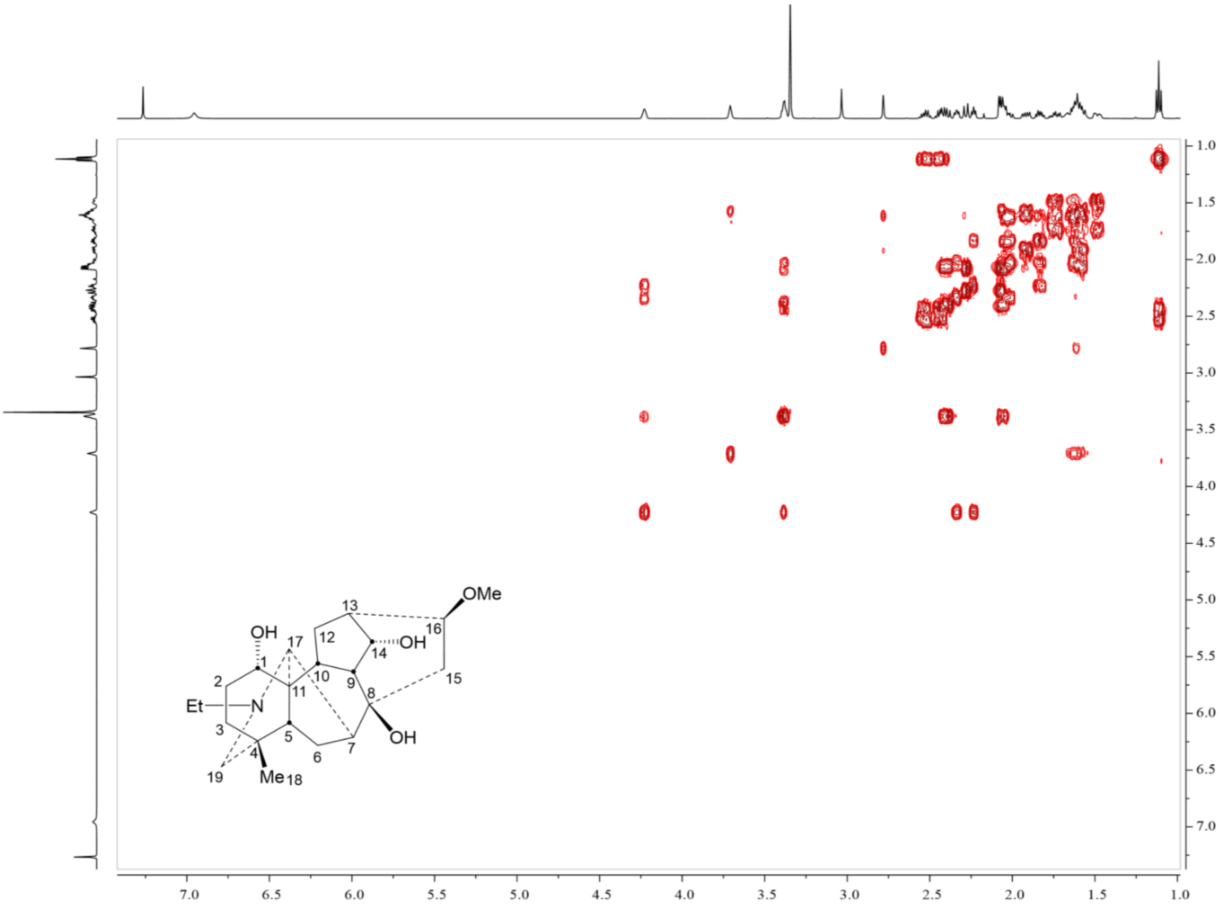

Figure S10. COSY spectrum of karacoline (**6**)

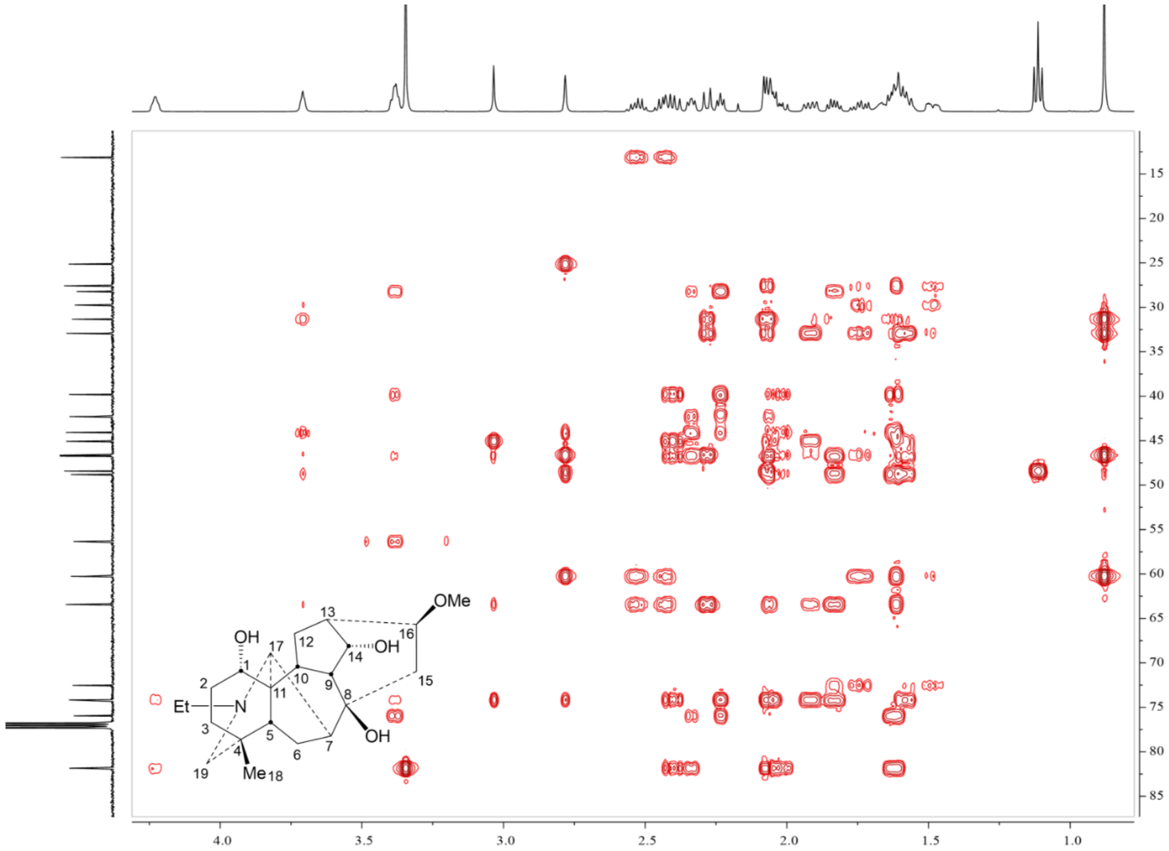

Figure S11. HMBC spectrum of karacoline (**6**)

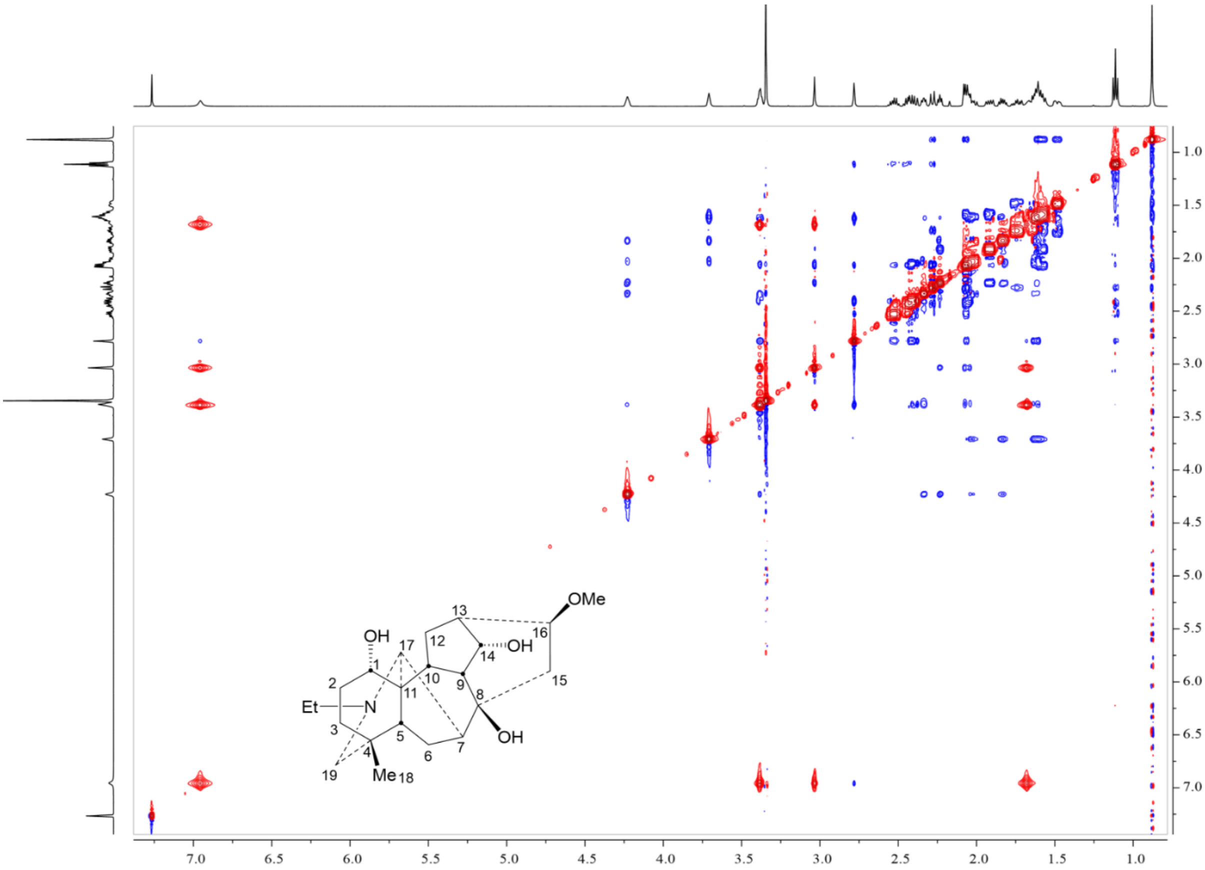

Figure S12. NOESY spectrum of karacoline (**6**)

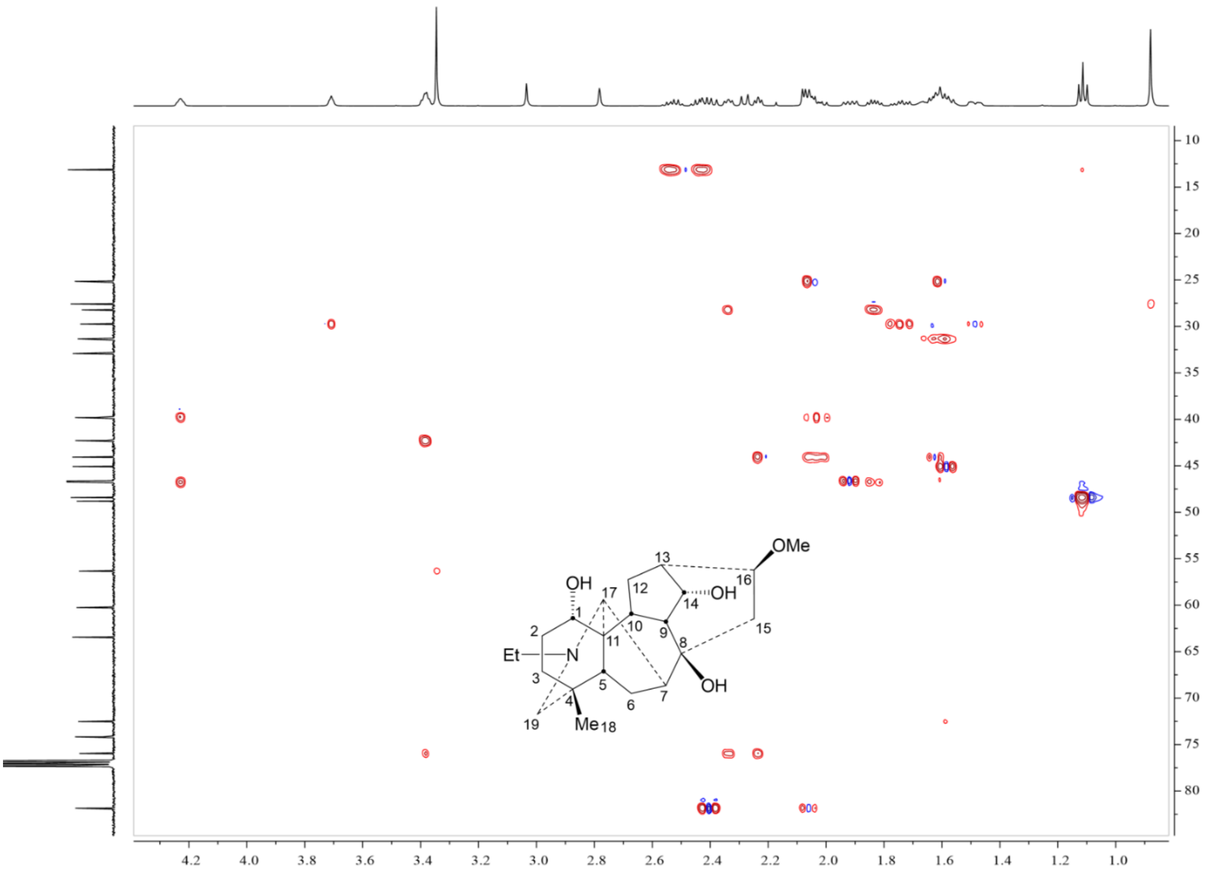

Figure S13. H2BC spectrum of karacoline (**6**)

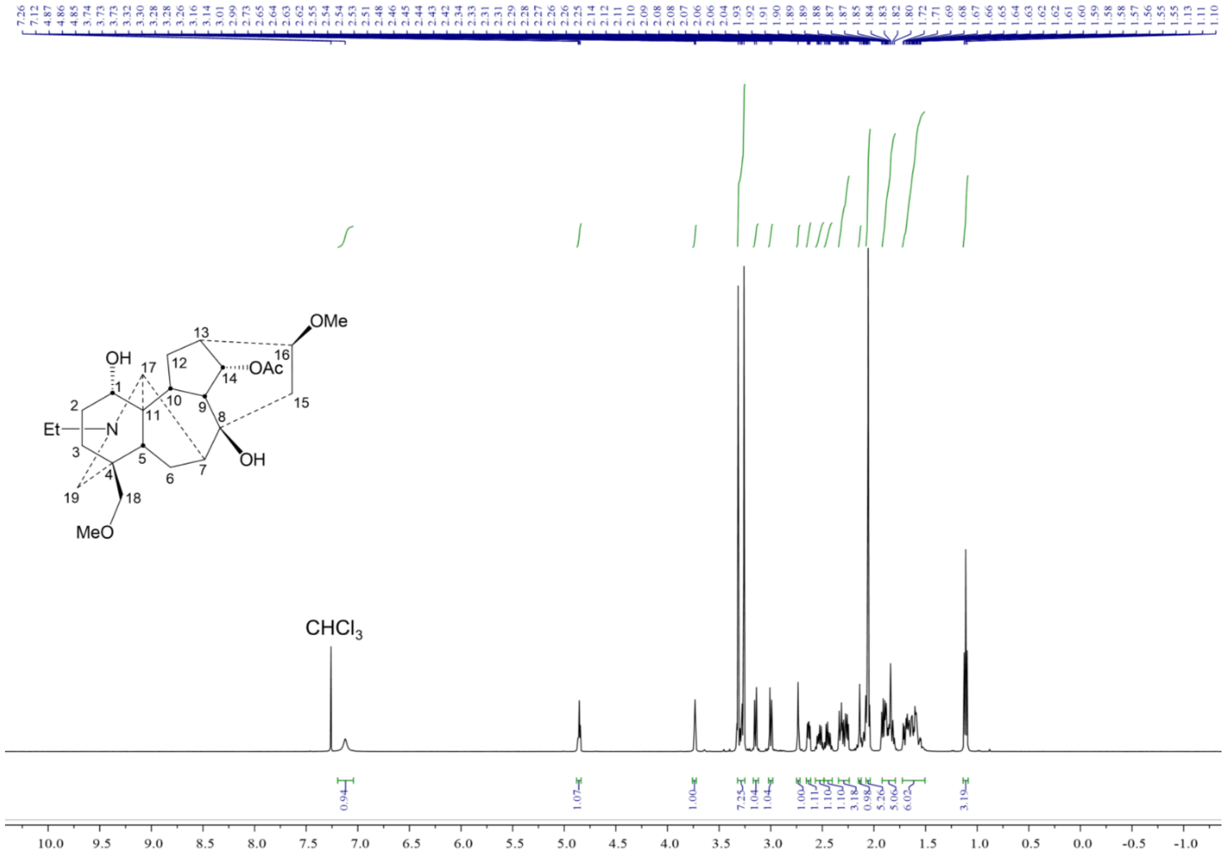

Figure S14. ^{1}H NMR spectrum of condelphine (**7**)

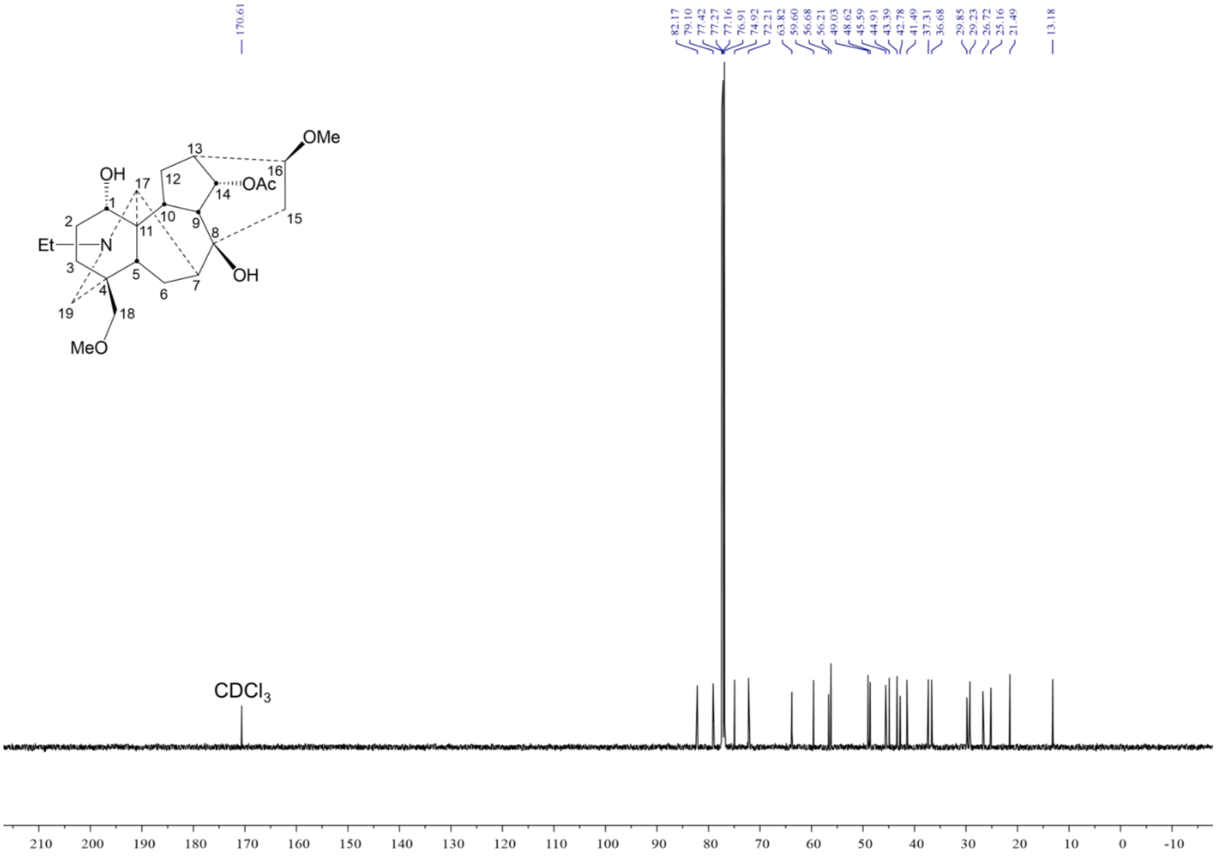

Figure S15. ^{13}C NMR spectrum of condelphine (**7**)

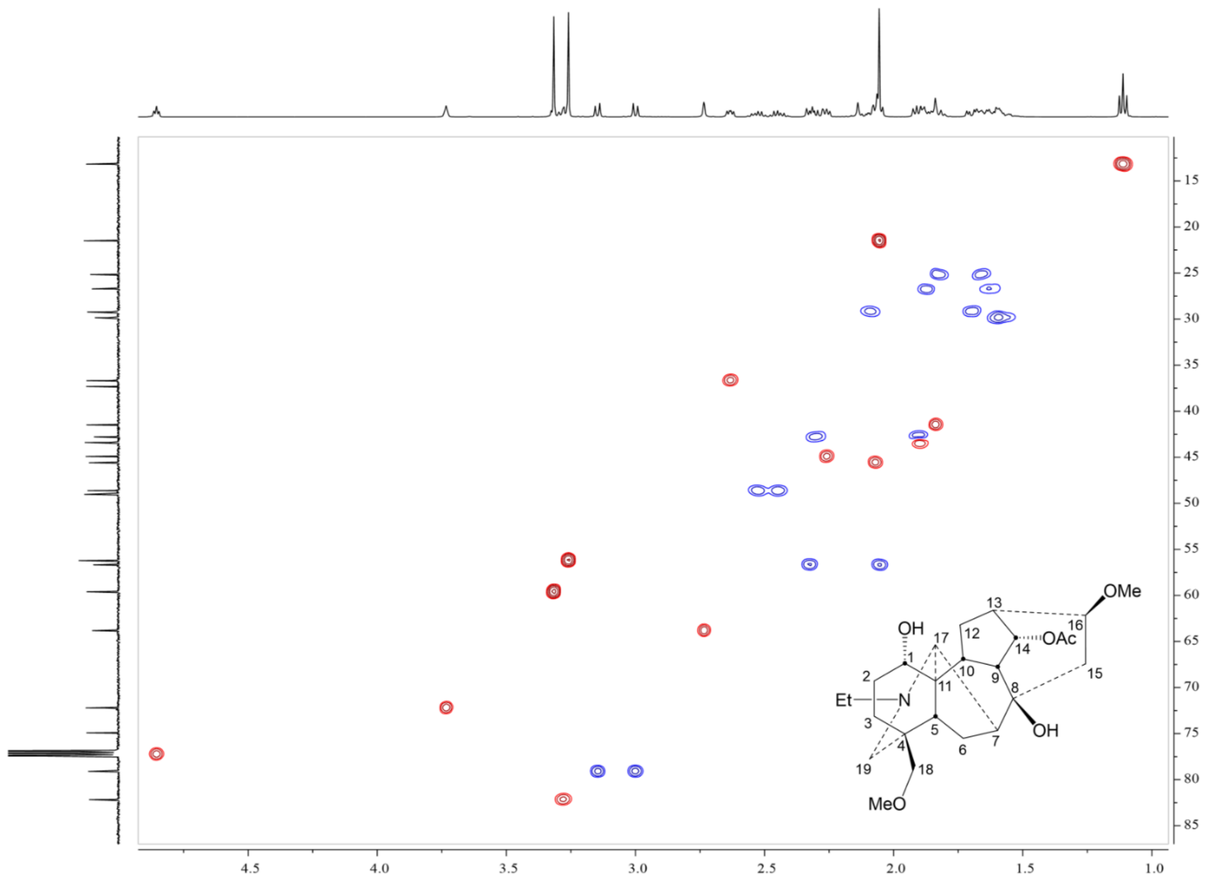

Figure S16. HSQC spectrum of condelphine (**7**)

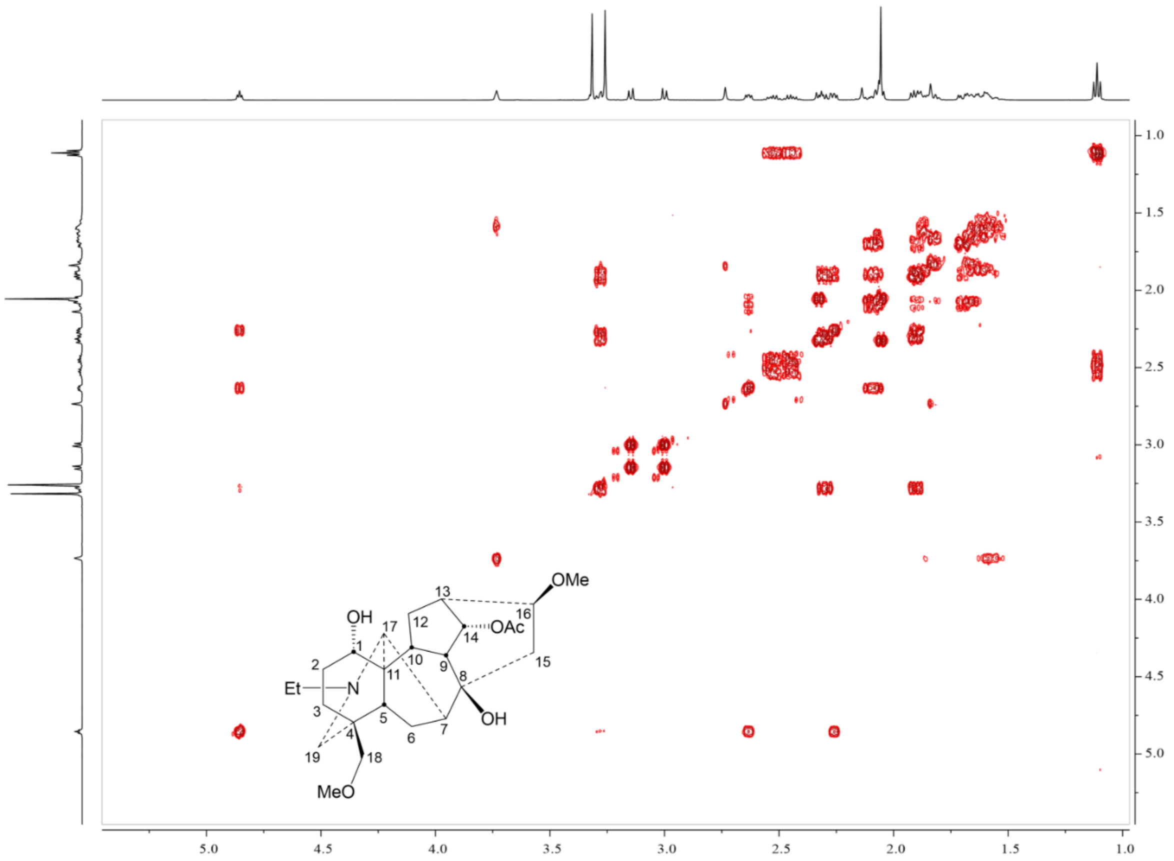

Figure S17. COSY spectrum of condelphine (**7**)

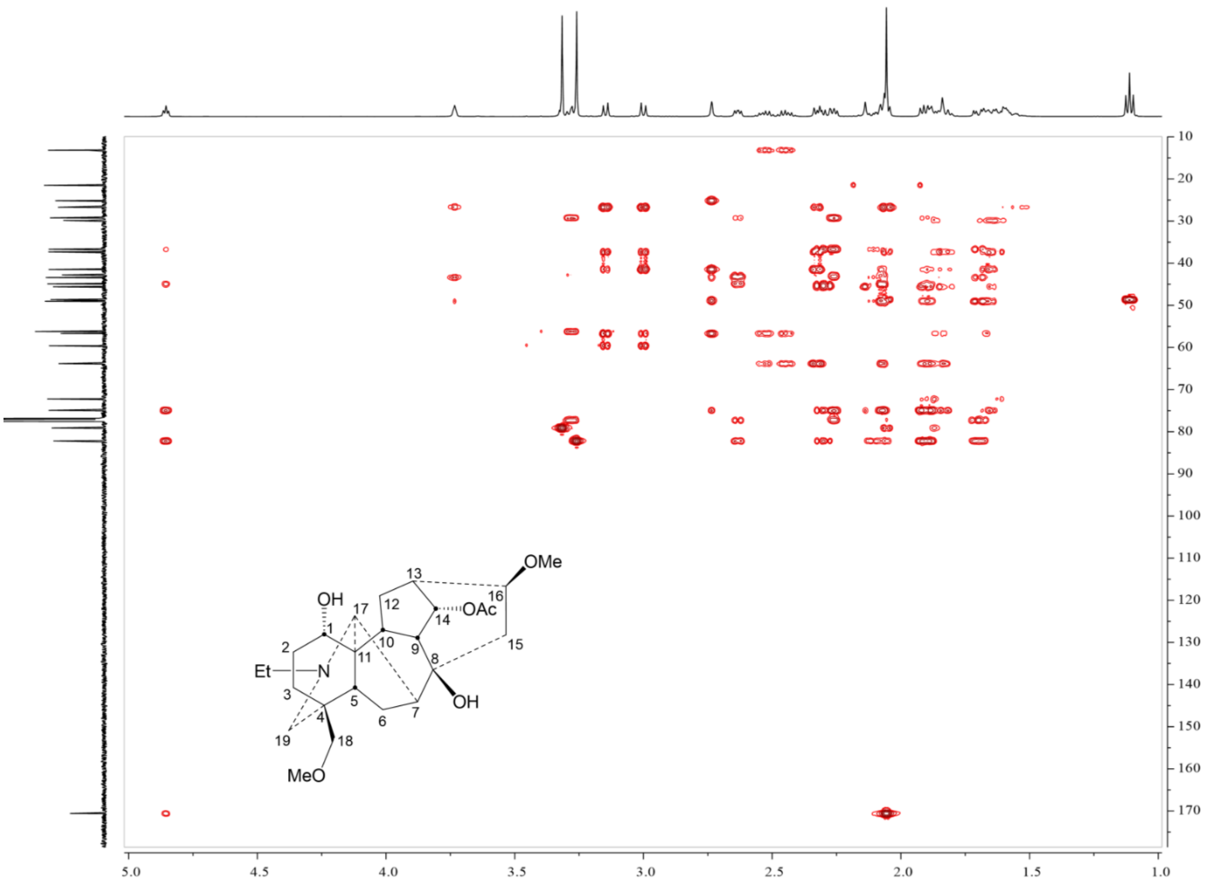

Figure S18. HMBC spectrum of condelphine (**7**)

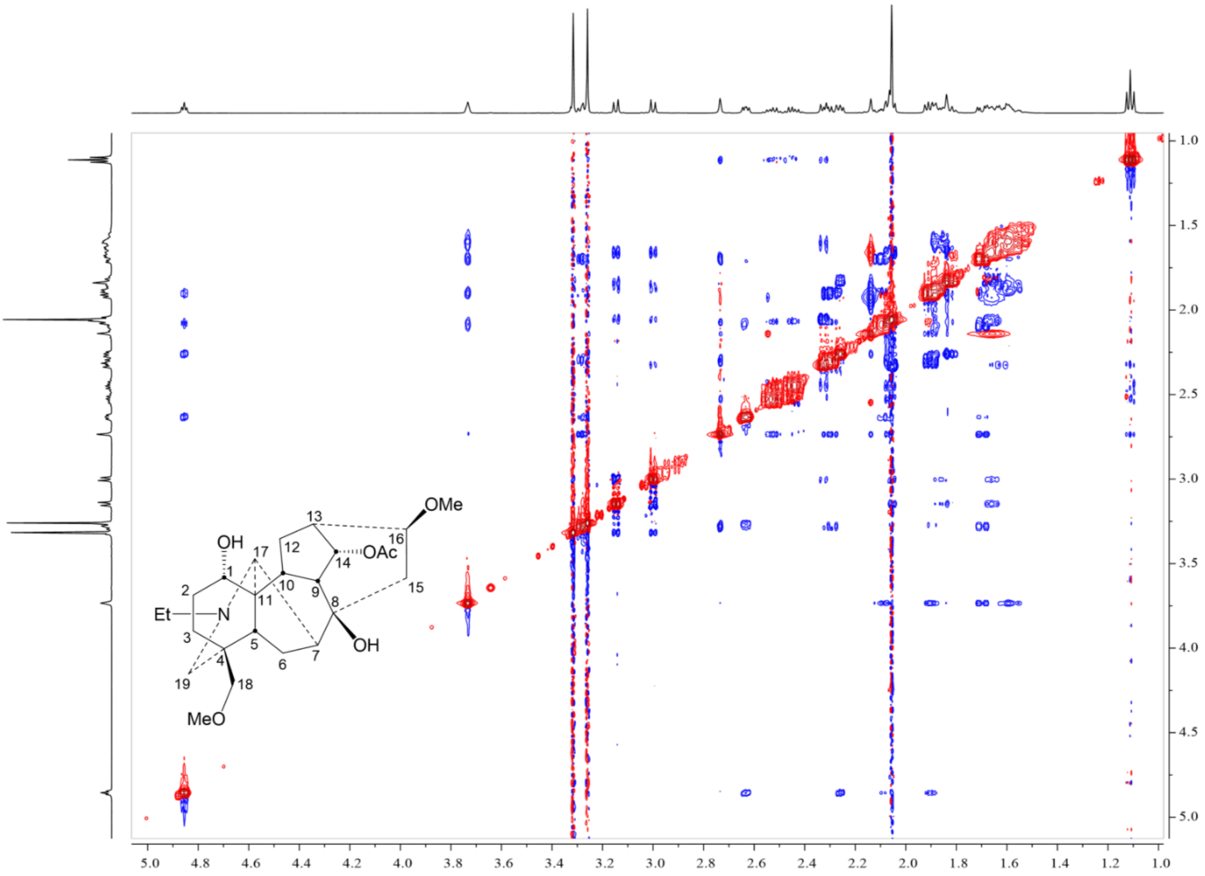

Figure S19. NOESY spectrum of condelphine (**7**)

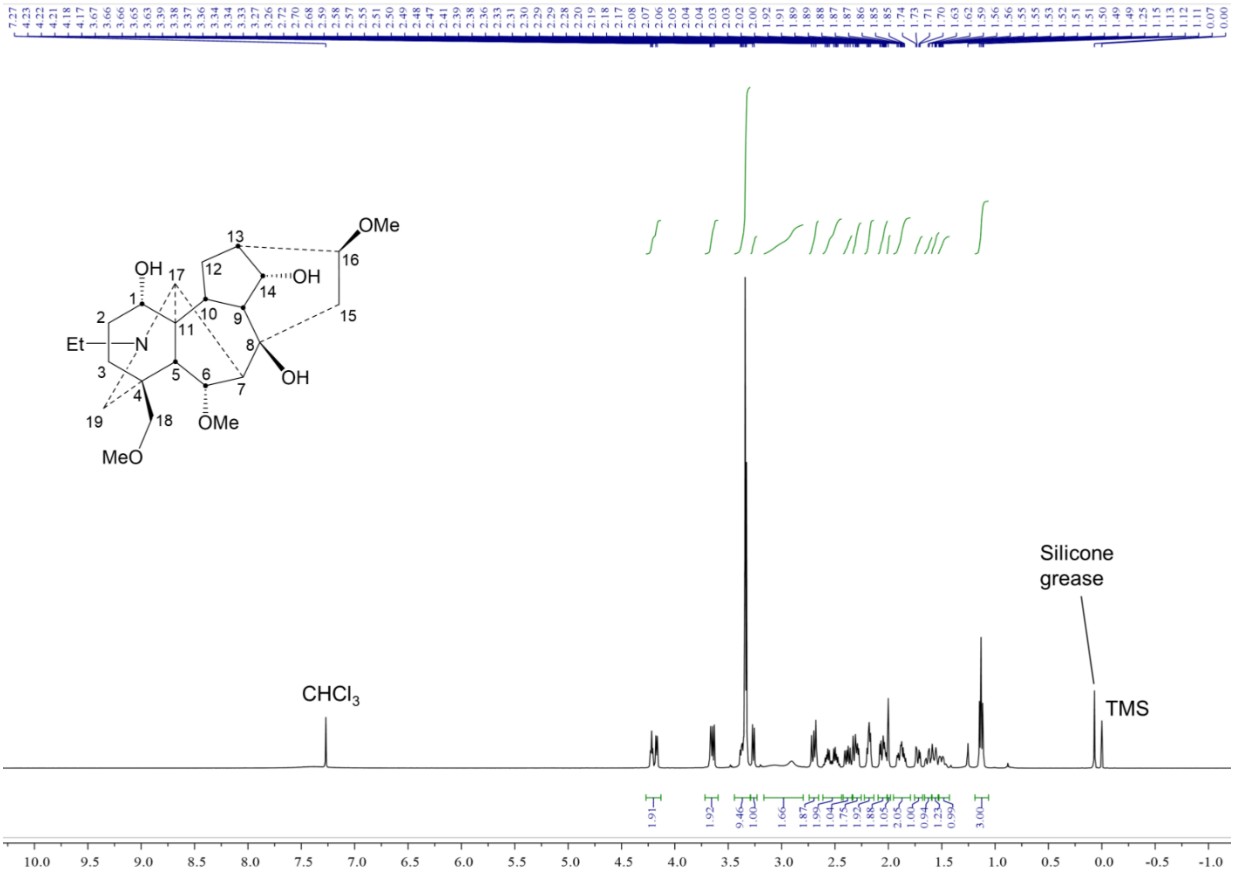

Figure S20. ^{1}H NMR spectrum of neoline (**8**)[8]

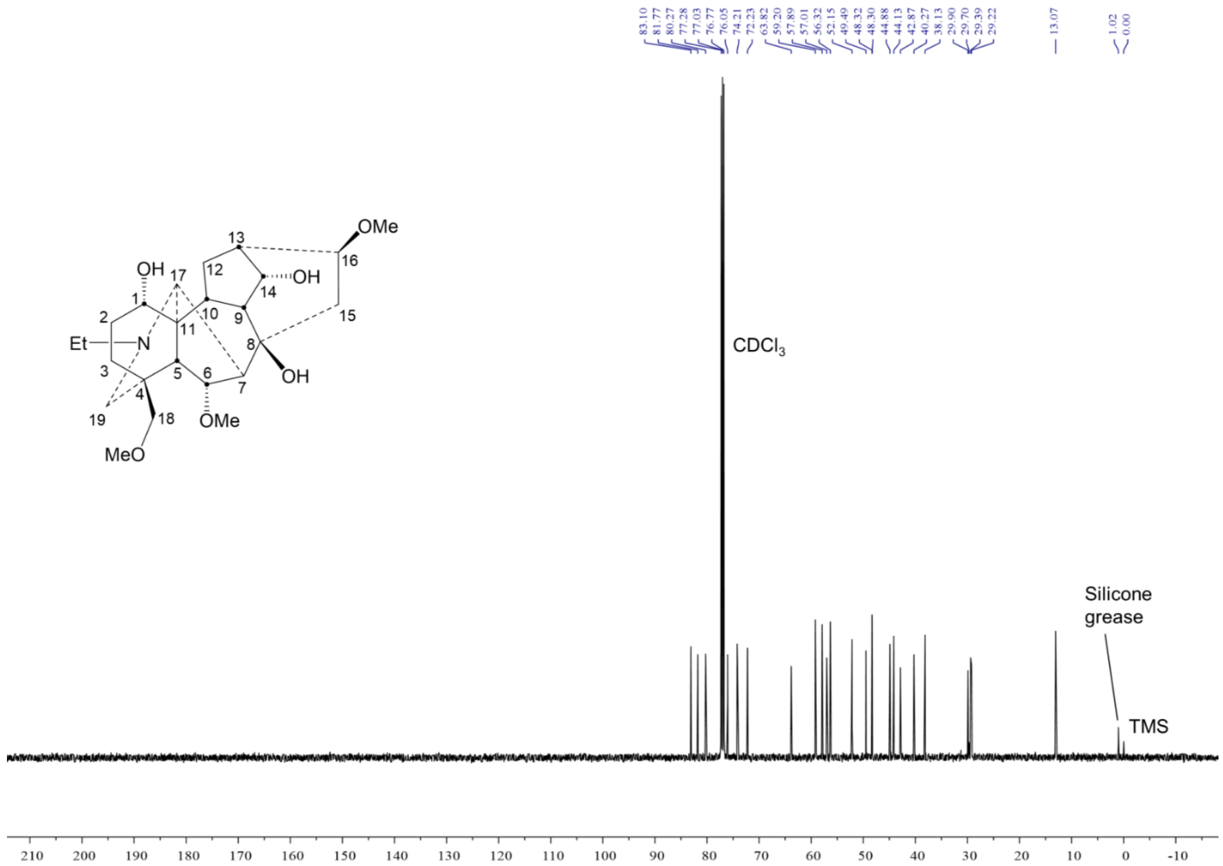

Figure S21. ^{13}C NMR spectrum of neoline (**8**)[8]

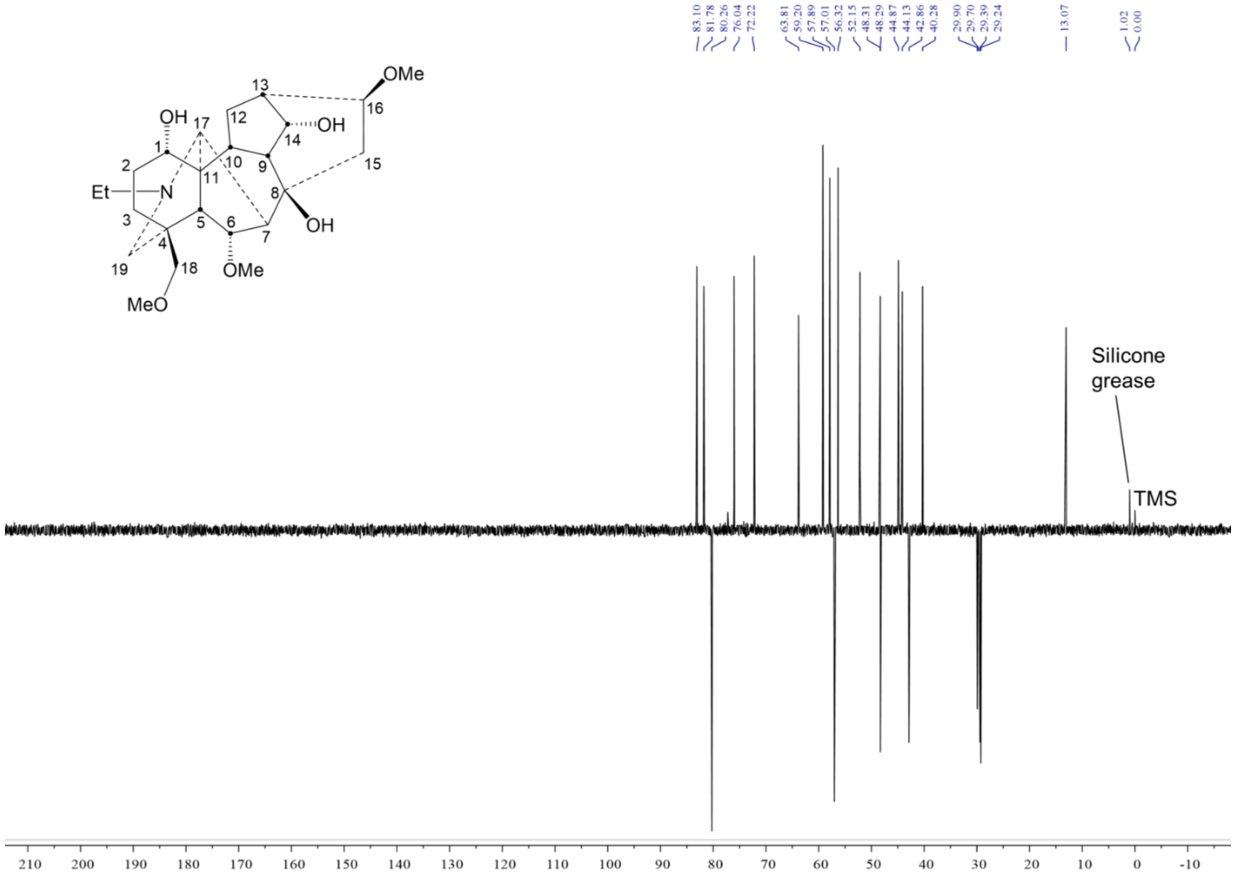

Figure S22. DEPT spectrum of neoline (**8**) (135°, no quat, CH ↑, CH_2 ↓, CH_3 ↑)

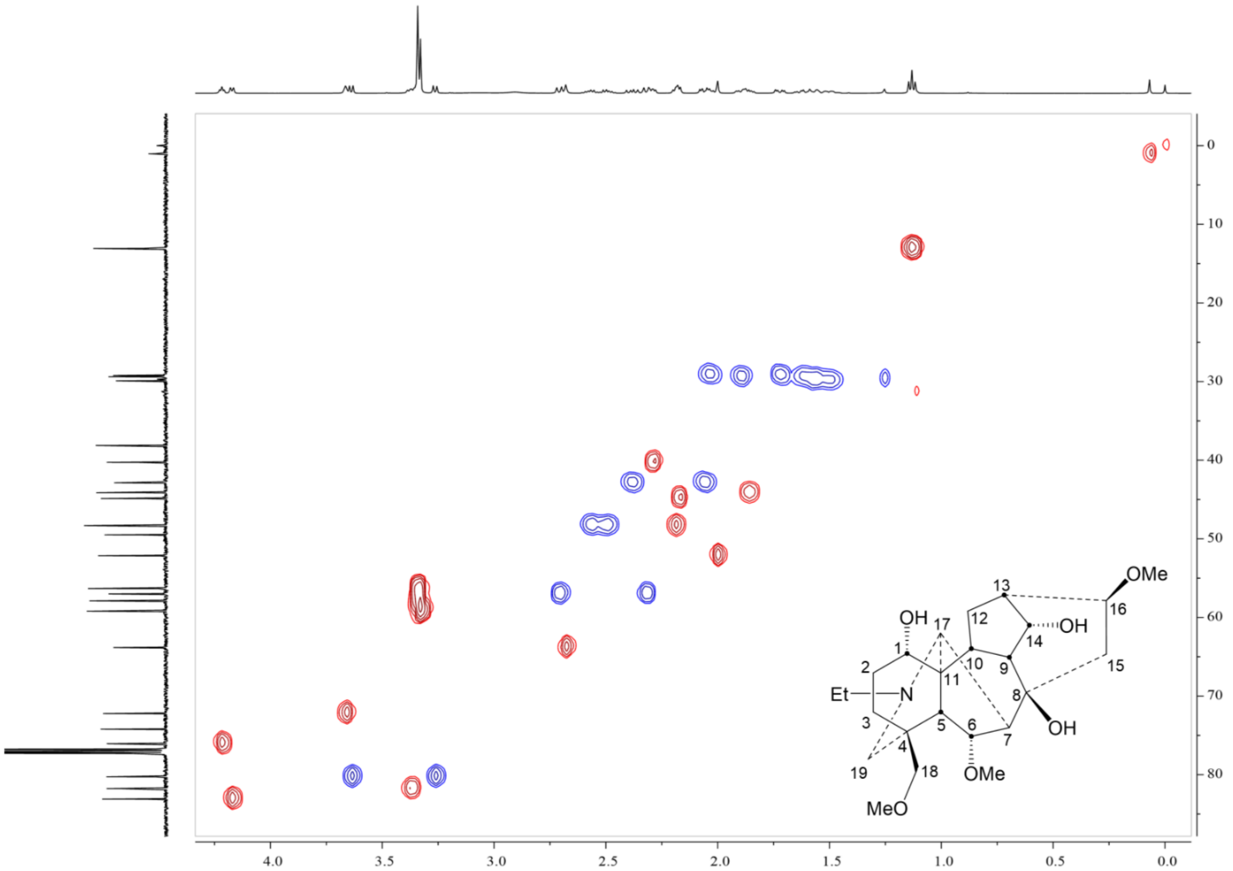

Figure S23. HSQC spectrum of neoline (**8**)

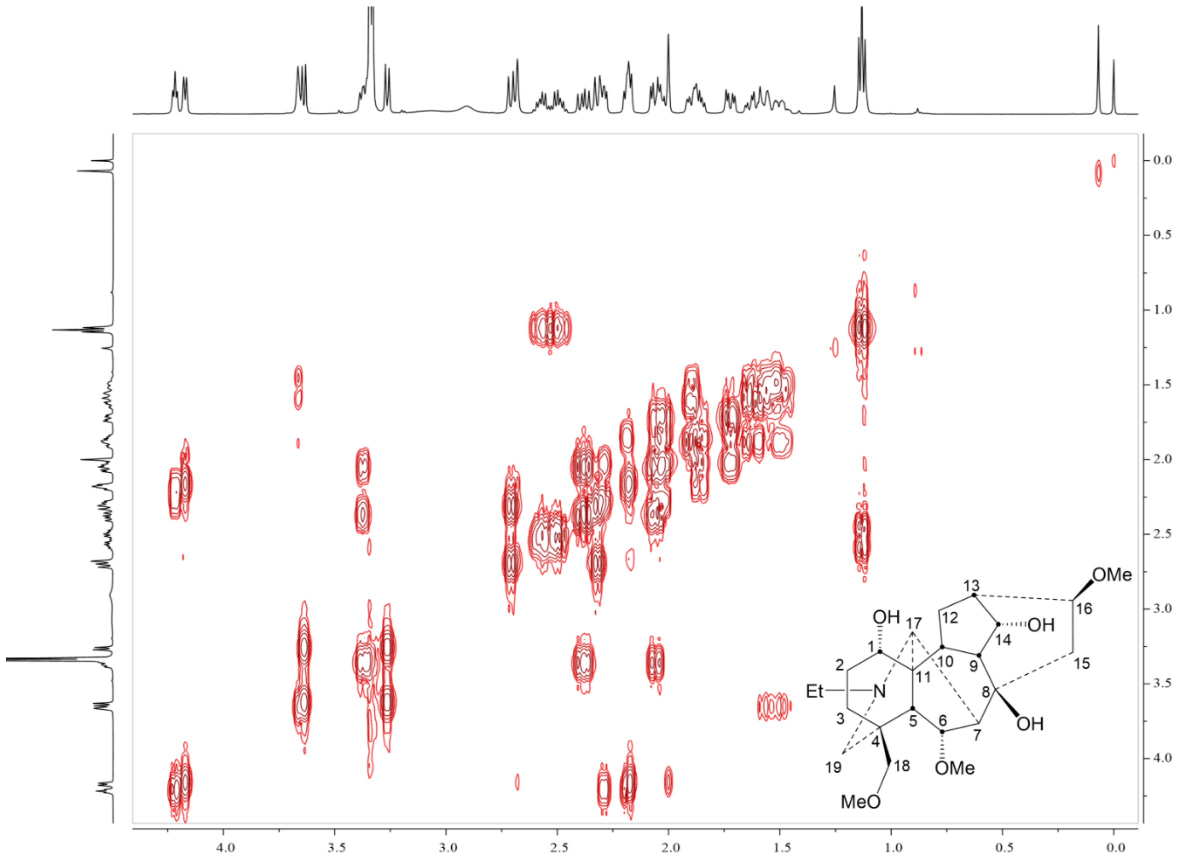

Figure S24. COSY spectrum of neoline (**8**)

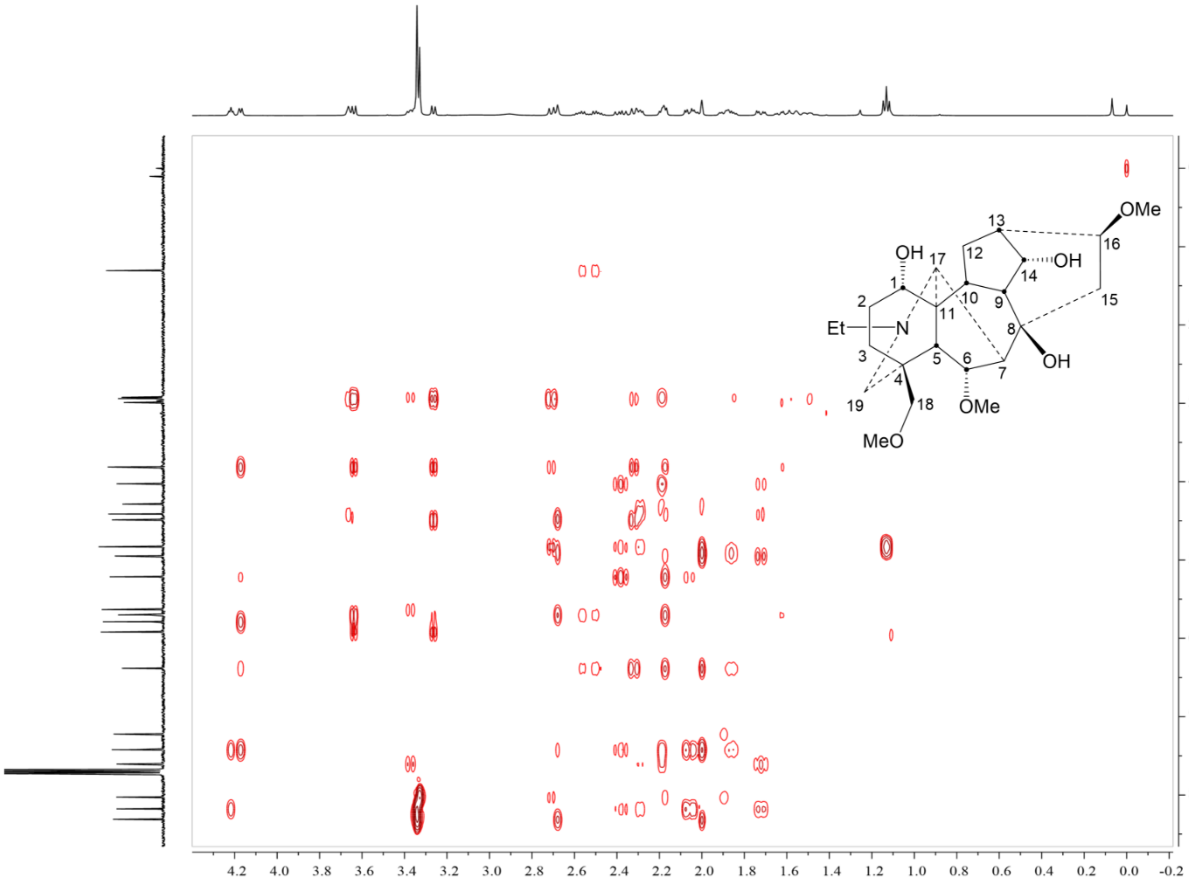

Figure S25. HMBC spectrum of neoline (**8**)

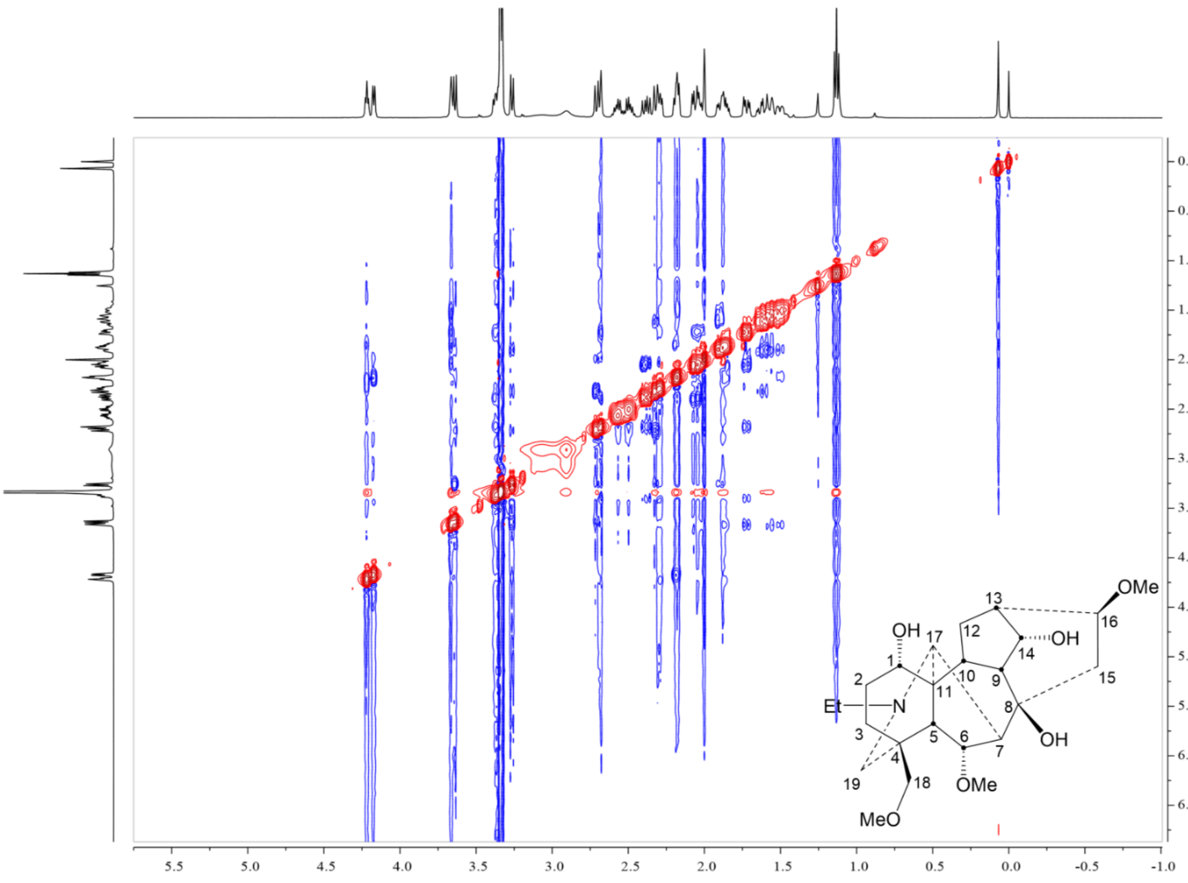

Figure S26. NOESY spectrum of neoline (**8**)

References

1 S. W. Pelletier and Z. Djarmati, *J. Am. Chem. Soc.*, 1976, **98**, 2626-2636.

2 K. Ishimi, M. Makino, Y. Asada, Y. Ichinohe and Y. Fujimoto, *J. Nat. Med.*, 2006, **60**, 255-257.

3 A. Ulubelen, H. K. Desai, S. K. Srivastava, B. P. Hart, J.-C. Park, B. S. Joshi, S. W. Pelletier, A. H. Meriçli, F. Meriçli and R. Ilarslan, *J. Nat. Prod.*, 1996, **59**, 360-366.

4 S. Sakai, H. Takayama and T. Okamoto, *Yakugaku Zasshi*, 1979, **99**, 647-656.

5 J. B. Hanuman and A. Katz, *Phytochemistry*, 1994, **36**, 1527-1535.

6 CrysAlisPro 1.171.40.43a (Rigaku Oxford Diffraction, 2019)

7 G. M. Sheldrick, *Acta Crystallogr. C*, 2015, **71**, 3-8.

8 H. E. Gottlieb, V. Kotlyar and A. Nudelman, *J. Org. Chem.*, 1997, **62**, 7512-7515.
